# Supplementary material for: Low dose cisplatin weekly versus high dose cisplatin every three weeks in primary chemoradiotherapy in head and neck cancer patients with low skeletal muscle mass: The CISLOW-study protocol
Source: PLoS One. 2023 Nov 27;18(11):e0294147. doi: 10.1371/journal.pone.0294147 (PMC10681175; doi:10.1371/journal.pone.0294147)
Supplement: S1 Checklist — (DOCX) [file pone.0294147.s001.docx]

##
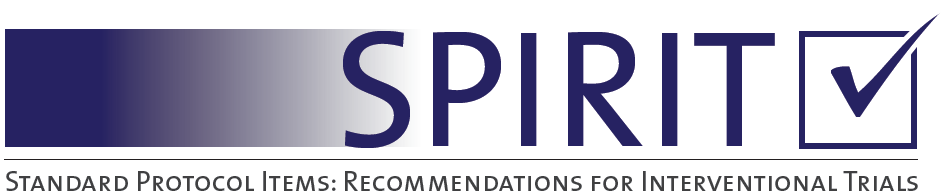


SPIRIT 2013 Checklist: Recommended items to address in a clinical trial protocol and related documents*

| Section/item | ItemNo | Description |
| --- | --- | --- |
| **Administrative information** | | |
| Title | 1 | Low dose cisplatin weekly versus high dose cisplatin every three weeks in primary chemoradiation in sarcopenic head and neck cancer patients (CISLOW) |
| Trial registration | 2a | NL76533.041.21, registered in the Netherlands Trial Register |
|  | 2b | Included |
| Protocol version | 3 | 20-02-2023 version 6.0 |
| Funding | 4 | ZonMw, projectnumber 10140021910002 |
| Roles and responsibilities | 5a | Authors: Drs. A.W.M.A. Schaeffers^1^, dr. L.A. Devriese^2^, prof. dr. C.H. van Gils^3^, dr. J.W. Dankbaar^4^, dr. J. Voortman^5^, dr. J.P. de Boer^6^, drs. E.J. Smid^7^, dr. G.W.J. Frederix^3^, Prof. dr. R. de Bree^1^  Affiliations:   1. Department of Head and Neck Surgical Oncology, University Medical Center Utrecht, Utrecht, The Netherlands 2. Department of Medical Oncology, University Medical Center Utrecht, Utrecht, The Netherlands 3. Julius Center for Health Sciences and Primary Care, University Medical Center Utrecht, Utrecht, The Netherlands 4. Department of Radiology, University Medical Center Utrecht, Utrecht, The Netherlands 5. Department of Medical Oncology, Amsterdam UMC, location VUmc, Cancer Center Amsterdam, Amsterdam, The Netherlands 6. Department of Medical Oncology, Antoni van Leeuwenhoek, Amsterdam, The Netherlands 7. Department of Radiotherapy, University Medical Center Utrecht, Utrecht, The Netherlands |
|  | 5b | Trial sponsor: University Medical Center Utrecht  Trial funding: The Netherlands Organisation for Health Research and Development (ZonMw), projectnumber 10140021910002  Contactname: Prof. dr. R. de Bree, principle investigator Adress: Huispost Q05.4.300 PO Box 85500 3508GA Utrecht Phone: 088-7550819 Email: [r.debree@umcutrecht.nl](mailto:r.debree@umcutrecht.nl) |
|  | 5c | Role of study sponsor in study design consist of collection, management, analysis, and interpretation of data; writing of the report; and the decision to submit the report for publication, and they will have ultimate authority over any of these activities. The funding institute approved the protocol during the grant application process. |
|  | 5d | The other centers assure inclusion and a researcher working at all five centers will collect data. Monitoring will be performed by an external monitoring company, hired by the UMCU. |
| Introduction |  |  |
| Background and rationale | 6a | In the Netherlands, 3080 patients were diagnosed with head and neck squamous cell carcinoma (HNSCC) in 2020.^1^ Two-thirds of HNSCC patients present with locally advanced HNSCC. The standard of care consists of intravenous cisplatin concurrently given with conventional external beam radiotherapy (chemoradiotherapy, CRT).^2–8^ High cumulative cisplatin dose is associated with better outcome.^9,10^ The most commonly used scheme is triweekly cisplatin of 100mg/m^2^. Though effective in terms of overall survival (OS) and loco-regional control (LRC), high rates of severe acute events lead to early cessation of therapy in up to 40% of patients and cause decrease in local control and survival.^11–13^ Furthermore in 13% of the patients late toxicity is reported, which leads to permanent comorbidity.^13^ Currently, patients at risk for this toxicity cannot be accurately identified upfront. Another commonly used scheme is weekly cisplatin of 40 mg/m2 concurrently given with radiotherapy (RT). In practice, this scheme is also widely accepted as standard of care as it results in less acute toxicity even though the high level of evidence using meta-analysis prefers the triweekly scheme on a group level.^11,13–22^ Currently, it is assumed that, in a not yet identified specific subset of patients at risk for toxicity following the triweekly scheme, weekly concurrent cisplatin might be more appropriate, leading to better tolerance, less toxicities and to a higher cumulative cisplatin dose.^13^ It has been shown previously that patients with low skeletal muscle mass (SMM) were more than three times more likely to develop cisplatin dose limiting toxicity (CDLT) compared to patients with normal SMM (44.3% vs. 13.7%); consequently, compliance (no CDLT) rate to planned chemotherapy scheme was 55.7% and 86.3%.^23^ Moreover, patients with CDLT also had a lower OS.^23^ This leads to the hypothesis that particularly patients with low SMM may benefit from weekly cisplatin concurrent RT, leading to better compliance compared to the triweekly schedule. We hypothesize that in HNSCC patients with low SMM, receiving weekly cisplatin concurrent RT can improve compliance rate to planned chemotherapy scheme from 55.7% to 86.3%. |
|  | 6b | Explanation for choice of comparators: Patients with low SMM will be randomized between chemotherapy treatment of either weekly cisplatin 40 mg/m^2^ body surface area and triweekly 100 mg/m^2^ cisplatin. The control group consists of low SMM patients receiving triweekly 100 mg/m2 cisplatin. An additional control group consists of normal SMM patients receiving local standard of care. |
| Objectives | 7 | To investigate if the use of weekly cisplatin increases compliance to the planned chemotherapy scheme in HNSCC patients with low SMM to a level of compliance to triweekly dose cisplatin of patients without low SMM. |
| Trial design | 8 | When the patient has signed informed consent, patients with low SMM will be randomised by the investigator for triweekly (three cycles 100mg/m^2^) or weekly cisplatin (seven cycles 40mg/m^2^). Allocation is performed by a central office on-site computer combined with allocations kept in a locked, unreadable computer file that investigators can assess only after the characteristics of an enrolled patients are entered. A stratified permuted-block procedure will randomise patients to the groups on a 1:1 ratio. Strata comprises centre and two groups according to UJCC staging:  1) Stage I-III  2) Stage IV.  Neither patients, investigators nor office personnel are blinded to the group chosen by the allocation procedure. |
| Methods: Participants, interventions, and outcomes | | |
| Study setting | 9 | Study setting will be in academic and cancer-specialized hospitals part of the Dutch Head and Neck Cancer Society (NWHHT). If additional centers participate this will be mentioned in the trial register. |
| Eligibility criteria | 10 | The intervention will be performed by the treating oncologist who will prescribe the treatment schedule.  Inclusion criteria:  - considered, eligible and planned for primary cisplatin CRT by treating physician;  - eighteen years of age or older;  - sufficient understanding of Dutch and medical consequences to give informed consent.  Exclusion criteria:  - mentally disabled or patients with significantly altered mental status that would prohibit understanding and giving informed consent;  - bilateral lymph node dissection in the neck and no available (PET-)CT scan of the third lumbar vertebra;  - an absolute contraindication for cisplatin as defined by the treating physician, including relevant pre-existing kidney insufficiency, clinically apparent vascular disease (for example claudicatio intermittens), clinically relevant perceptive deafness, serious neuropathy and poor performance score;  - an absolute contraindication for triweekly cisplatin 100 mg/m2 as defined by the treating physician;  - interval between diagnostic scan and planned CRT >2 months;  - cisplatin CRT planned as non-primary or induction treatment. |
| Interventions | 11a | Patients with low SMM are randomised for triweekly (three cycles of 100mg/m^2^) or weekly (seven cycles of 40mg/m^2^) cisplatin intravenously given with concurrent radiotherapy (35x 2 Gy; five times weekly with a total of 7 weeks). |
|  | 11b | The treating oncologist decides whether the treatment is continued, postponed or stopped. Participation in the study has no influence in this decision making. |
|  | 11c | Not applicable, the patient receives the medicine intravenously so control will be done by automated processes. |
|  | 11d | Everything, except the randomisation for the schedule, is conform standard of care. No additional treatment, or excepted treatments are part of the study. |
| Outcomes | 12 | The primary outcome of this study is the difference in compliance (defined as absence of CDLT) rate to the proposed cisplatin scheme between weekly cisplatin and triweekly cisplatin in patients with low SMM. Secondary outcomes of this study are treatment cumulative cisplatin dose, acute and late adverse events (AE’s), time to recurrence, 2-years survival, quality of life, costs and patient’s preference. Toxicities are reported according to the Common Terminology Criteria of Adverse Events (CTCAE) version 5.0 criteria^12^, because this is often used in oncology. The other parameters are chosen by extensively reading other research and our goal to make the research as reproducible as possible. |
| Participant timeline | 13 | A schematic diagram is shown in Figure 1 |
| Sample size | 14 | In the retrospective study by Wendrich et al., out of 112 patients 30.4% experienced CDLT (meaning compliance to chemotherapy regimen was 69.6%). Using a cut-off 43.2 cm2/m2, 54.5% patients had low SMM. Patients with low SMM experienced CDLT more frequently than patients with normal SMM (44.3% vs. 13.7%, p < 0.001).^23^ Thus compliance rates to chemotherapy regimen were 55.7% and 86.3%, respectively. We hypothesize therefore that in patients with low skeletal muscle mass and weekly cisplatin, also 86.3% compliance will be reached. If we compare this compliance to an expected compliance of 55.7% in the arm of with a triweekly cisplatin, 33 patients with low SMM per arm are needed to show that the compliance in the weekly cisplatin scheme is statistically significantly better than the triweekly scheme (two-sided alpha 0.05 and power 80%). With an expected drop-out of 5% 70 patients with low SMM are needed. Power calculations were done using PASS software. Since 54.5% of patients undergoing cisplatin based concurrent CRT have low SMM, to find the 70 patients with low SMM a total of 129 HNSCC patients undergoing cisplatin based concurrent CRT are needed. |
| Recruitment | 15 | All patients diagnosed with an indication for primary CRT, will be identified and screened for eligibility at a weekly multidisciplinary head and neck oncology meeting. The researcher, specialized nurse, research nurse or oncologist will inform the patient about the study. |
| **Methods: Assignment of interventions (for controlled trials)** | | |
| Allocation: |  |  |
| Sequence generation | 16a | A stratified permuted-block procedure will randomise patients to the groups on a 1:1 ratio. Strata comprises centre and two groups according to UJCC staging:  1) Stage I-III  2) Stage IV. |
| Allocation concealment mechanism | 16b | Castor EDC will be used for randomisation which is an independent tool for researchers to perform randomisation. |
| Implementation | 16c | The allocation sequence is created by Castor EDC. The patients are enrolled by a research, oncologist, research nurse or (specialized) nurse. |
| Blinding (masking) | 17a | Blinding is not done. |
|  | 17b | Not applicable |
| **Methods: Data collection, management, and analysis** | | |
| Data collection methods | 18a | Castor EDC is used for data management and it is built so changes can be tracked and it promotes data quality by having limits for number-entries. Questionnaires are already validated for use. Laboratory tests are part of standard of care and done by a certified The case report form can be found as supplement S4 |
|  | 18b | Follow-up is done according to standard of care so promotion of follow-up is not necessary. Every three months electronic patient records are used to collect data. |
| Data management | 19 | Data entry and coding is done via Castor EDC. Security is assured using the protected drives from the UMCU, Amsterdam UMC location VUmc and AVL. Storage will be done on these drives or, after termination of the study and during the required time of data-saving, at an external location for data storage for that specific hospital if necessary. See the data management plan S3 |
| Statistical methods | 20a | Categorical data will be represented as a number and percentage of the total. Data analysis will be performed using statistical software R and SPSS Statistics. A test for normality (Kolmogorov-Smirnoff test) and histograms will be used to assess whether continuous variables are normally distributed. Continuous data will be represented as mean ± standard deviation (SD) if normally distributed, and median ± interquartile range (IQR) if skewed. Fisher’s exact tests, Pearson Chi square tests, independent sample t-tests and Mann-Whitney U tests will be used to assess group differences. All analyses will be two-sided and p<0.05 is considered significant. |
|  | 20b | Any additional analyses will also be done conform 20a. 2-years survival and recurrence will be analysed using Kaplan-Maier and Cox regression analysis. |
|  | 20c | Missing data will be handled using multiple imputation, if needed; we only expect few missing data to occur due to the nature and scale of the study. |
| **Methods: Monitoring** | | |
| Data monitoring | 21a | Monitoring is guaranteed by the UMC Utrecht by a centralised, independent monitor. The monitoring plan for the research will be added as S2. |
|  | 21b | There are no interim analyses planned. In accordance to section 10, subsection 4, of the WMO, the sponsor will suspend the study if there is sufficient ground that continuation of the study will jeopardise subject health or safety. The sponsor will notify the ethical committee of the UMCU without undue delay of a temporary halt including the reason for such an action. The study will be suspended pending a further positive decision by the ethical committee of the UMCU. The investigator will take care that all subjects are kept informed. |
| Harms | 22 | Adverse events, serious adverse events and suspected unexpected serious adverse reactions are reported conform the study protocol and can be read in the original study protocol submitted to the ethical committee. |
| Auditing | 23 | No auditing is planned, it is possible that an audit is performed based on random auditing of studies in the UMCU. |
| Ethics and dissemination | | |
| Research ethics approval | 24 | METC number NL76533.041.21 |
| Protocol amendments | 25 | Not applicable at the moment |
| Consent or assent | 26a | Differs per centre, in the UMCU the executive researcher asks, in the other centres either the oncologist, the research-nurse or specialized nurse will ask the patient for consent. |
|  | 26b | Not applicable |
| Confidentiality | 27 | All patients will receive pseudonyms and these pseudonyms will be used for data analysis. In case of a data transfer, the sponsor first has to approve this and contracts have to be created and signed by both parties. |
| Declaration of interests | 28 | No competing interests to declare. |
| Access to data | 29 | Data will be coded according the data management plan. The principal investigator, the study team, data management and monitor will have access to the source data. The key to the code will be safeguarded by the study coordinators. Data will be kept 15 years. A web-based data management system (Castor EDC) will be used for data management. For exact details, please see the Datamanagement plan in supplement S4. |
| Ancillary and post-trial care | 30 | There is a subject trial and a liability insurance. |
| Dissemination policy | 31a | The publication will have open access. The study protocol and this Data Management Plan will also be available. |
|  | 31b | Authorship eligibility depends on amount of work the author put into the writing. There is no intended use of professional writers. |
|  | 31c | Our data will be shared with third parties after approval of the Principle Investigator. The criteria and time period will be determined on a case-by-case basis. |
| Appendices |  |  |
| Informed consent materials |  | See supplement S2 for an example in Dutch |
| Biological specimens Monitoring plan Data management plan  Case report form |  | Not applicable  See supplement S3  See supplement S4  Not applicable |

*It is strongly recommended that this checklist be read in conjunction with the SPIRIT 2013 Explanation & Elaboration for important clarification on the items. Amendments to the protocol should be tracked and dated. The SPIRIT checklist is copyrighted by the SPIRIT Group under the Creative Commons “[Attribution-NonCommercial-NoDerivs 3.0 Unported](http://www.creativecommons.org/licenses/by-nc-nd/3.0/)” license.

1. IKNL. Incidentie hoofd-halskanker. Available from: https://iknl.nl/kankersoorten/hoofd-halskanker/registratie/incidentie. Accessed on 23-03-2023.

2. Grégoire V, Lefebvre JL, Licitra L, Felip E. Squamous cell carcinoma of the head and neck: EHNS-ESMO-ESTRO clinical practice guidelines for diagnosis, treatment and follow-up. *Ann Oncol*. 2010;21(SUPPL. 5):184-186. doi:10.1093/annonc/mdq185

3. Adelstein DJ, Li Y, Adams GL, et al. An intergroup phase III comparison of standard radiation therapy and two schedules of concurrent chemoradiotherapy in patients with unresectable squamous cell head and neck cancer. *J Clin Oncol*. 2003;21(1):92-98. doi:10.1200/JCO.2003.01.008

4. Forastiere AA, Goepfert H, Maor M, et al. Concurrent chemotherapy and radiotherapy for organ preservation in advanced laryngeal cancer. *N Engl J Med*. 2003;349(22):2091-2098. doi:10.1056/NEJMoa031317

5. Sher DJ, Adelstein DJ, Bajaj GK, et al. Radiation therapy for oropharyngeal squamous cell carcinoma: Executive summary of an ASTRO Evidence-Based Clinical Practice Guideline. *Pract Radiat Oncol*. 2017;7(4):246-253. doi:10.1016/j.prro.2017.02.002

6. Oosting SF, Haddad RI. Best practice in systemic therapy for head and neck squamous cell carcinoma. *Front Oncol*. 2019;9(AUG):1-9. doi:10.3389/fonc.2019.00815

7. Machiels JP, René Leemans C, Golusinski W, Grau C, Licitra L, Gregoire V. Squamous cell carcinoma of the oral cavity, larynx, oropharynx and hypopharynx: EHNS–ESMO–ESTRO Clinical Practice Guidelines for diagnosis, treatment and follow-up†. *Ann Oncol*. 2020;31(11):1462-1475. doi:10.1016/j.annonc.2020.07.011

8. Gebre-Medhin M, Brun E, Engström P, et al. ARTSCAN III: A randomized phase III study comparing chemoradiotherapy with cisplatin versus cetuximab in patients with locoregionally advanced head and neck squamous cell cancer. *J Clin Oncol*. 2021;39(1):38-47. doi:10.1200/JCO.20.02072

9. Pignon JP, Maître A le, Maillard E, Bourhis J. Meta-analysis of chemotherapy in head and neck cancer (MACH-NC): An update on 93 randomised trials and 17,346 patients. *Radiother Oncol*. 2009;92(1):4-14. doi:10.1016/j.radonc.2009.04.014

10. Calais G, Alfonsi M, Bardet E, et al. Randomized Trial of Radiation Therapy Versus. *J Natl Cancer Inst*. 1999;91(24):2081-2086.

11. Ghosh S, Rao PB, Kumar PR, Manam S. Concurrent chemoradiation with weekly cisplatin for the treatment of head and neck cancers: An institutional study on acute toxicity and response to treatment. *Asian Pacific J Cancer Prev*. 2015;16(16):7331-7335. doi:10.7314/APJCP.2015.16.16.7331

12. National Cancer Institute. US Department of Health and Human Services. Common Terminology Criteria for Adverse Events Version 5.0.

13. Szturz P, Wouters K, Kiyota N, et al. Low-dose vs. high-dose cisplatin: Lessons learned from 59 chemoradiotherapy trials in head and neck cancer. *Front Oncol*. 2019;9(FEB). doi:10.3389/fonc.2019.00086

14. Sharma A, Mohanti BK, Thakar A, Bahadur S, Bhasker S. Concomitant chemoradiation versus radical radiotherapy in advanced squamous cell carcinoma of oropharynx and nasopharynx using weekly cisplatin: A phase II randomized trial. *Ann Oncol*. 2010;21(11):2272-2277. doi:10.1093/annonc/mdq219

15. National Comprehensive Cancer Network. NCCN Guidelines Version 1.2021 Head and neck cancers.

16. Ho KF, Swindell R, Brammer C V. Dose intensity comparison between weekly and 3-weekly Cisplatin delivered concurrently with radical radiotherapy for head and neck cancer: A retrospective comparison from New Cross Hospital, Wolverhampton, UK. *Acta Oncol (Madr)*. 2008;47(8):1513-1518. doi:10.1080/02841860701846160

17. Gupta T, Agarwal JP, Ghosh-Laskar S, Parikh PM, D’Cruz AK, Dinshaw KA. Radical radiotherapy with concurrent weekly cisplatin in loco-regionally advanced squamous cell carcinoma of the head and neck: a single-institution experience. *Head Neck Oncol*. 2009;1:17. doi:10.1186/1758-3284-1-17

18. Otty Z, Skinner MB, Dass J, et al. Efficacy and tolerability of weekly low-dose cisplatin concurrent with radiotherapy in head and neck cancer patients. *Asia Pac J Clin Oncol*. 2011;7(3):287-292. doi:10.1111/j.1743-7563.2011.01405.x

19. Rawat S, Srivastava H, Ahlawat P, et al. Weekly versus Triweekly Cisplatin-based Concurrent Chemoradiotherapy as definitive treatment in Head and Neck Cancer- Where do we stand? *Gulf J Oncolog*. 2016;1(21):6-11.

20. Traynor AM, Richards GM, Hartig GK, et al. Comprehensive IMRT plus weekly cisplatin for advanced head and neck cancer: the University of Wisconsin experience. *Head Neck*. 2010;32(5):599-606. doi:10.1002/hed.21224

21. Boulmay BC, Chera BS, Morris CG, et al. Definitive altered fractionation radiotherapy and concomitant weekly cisplatin for locally advanced head and neck cancer. *Am J Clin Oncol*. 2009;32(5):488-491. doi:10.1097/COC.0b013e318194f418

22. Laskar SG, Chaukar D, Deshpande M, et al. Phase III randomized trial of surgery followed by conventional radiotherapy (5 fr/Wk) (Arm A) vs concurrent chemoradiotherapy (Arm B) vs accelerated radiotherapy (6fr/Wk) (Arm C) in locally advanced, stage III and IV, resectable, squamous cell carcinoma o. *J Clin Oncol*. 2016;34(15_suppl):6004. doi:10.1200/JCO.2016.34.15_suppl.6004

23. Wendrich AW, Swartz JE, Bril SI, et al. Low skeletal muscle mass is a predictive factor for chemotherapy dose-limiting toxicity in patients with locally advanced head and neck cancer. *Oral Oncol*. 2017;71:26-33. doi:10.1016/j.oraloncology.2017.05.012
