# Supplementary material for: Low dose cisplatin weekly versus high dose cisplatin every three weeks in primary chemoradiotherapy in head and neck cancer patients with low skeletal muscle mass: The CISLOW-study protocol
Source: PLoS One. 2023 Nov 27;18(11):e0294147. doi: 10.1371/journal.pone.0294147 (PMC10681175; doi:10.1371/journal.pone.0294147)
Supplement: S1 File — (PDF) [file pone.0294147.s003.pdf]

Proefpersoneninformatie voor deelname aan medisch-wetenschappelijk onderzoek

Onderzoek naar het volbrengen van de geplande kuren cisplatinum bij hoofd-halskankerpatiënten met een lage skeletspiermassa door verschillende toedieningsschema's

Officiële titel: Wekelijks lage-dosis versus drie-wekelijks hoge-dosis cisplatinum chemoradiatie bij hoofd-halskankerpatiënten met lage skeletspiermassa (CISLOW-studie).

Inleiding

Geachte heer/mevrouw,

Met deze informatiebrief willen we u vragen of u wilt meedoen aan medisch-wetenschappelijk onderzoek. Meedoen is vrijwillig. U krijgt deze brief omdat bij u een lage skeletspiermassa en een tumor (kanker) in het hoofd-halsgebied is vastgesteld, waar u binnenkort voor behandeld wordt met een combinatiebehandeling van chemotherapie en radiotherapie.

U leest hier om wat voor onderzoek het gaat, wat het voor u betekent, en wat de voor- en nadelen zijn. Het is veel informatie. Wilt u de informatie doorlezen en beslissen of u wilt meedoen? Als u wilt meedoen, kunt u het formulier invullen dat u in tweevoud vindt in bijlage C. Deze mag u dan overhandigen aan de onderzoeker of per post terugsturen middels bijgevoegde envelop. U krijgt een exemplaar getekend retour, het andere wordt door het onderzoeksteam bewaard ter administratie.

Stel uw vragen

U kunt uw beslissing nemen met de informatie die u in deze informatiebrief vindt. Daarnaast raden we u aan om dit te doen:

- Stel vragen aan de onderzoeker die u deze informatie geeft.
- Praat met uw partner, familie of vrienden over dit onderzoek.
- Stel vragen aan de onafhankelijk deskundige, de contactgegevens vindt u onderaan deze informatiebrief.
- Lees de informatie op [www.rijksoverheid.nl/mensenonderzoek.](http://www.rijksoverheid.nl/mensenonderzoek)

1. Algemene informatie

Het UMC Utrecht heeft dit onderzoek opgezet. Hieronder noemen we het UMC Utrecht steeds de 'opdrachtgever'. Onderzoekers, dit kunnen artsen en onderzoekers zijn, voeren het onderzoek uit in verschillende ziekenhuizen, namelijk het UMC Utrecht, de Amsterdam UMC (locatie VUmc), de Noordwest Ziekenhuisgroep, Leiden Universitair Medisch Centrum en het Antoni van Leeuwenhoek Ziekenhuis. Voor dit onderzoek is een subsidie verkregen vanuit

ZonMw, dit is een organisatie voor onder andere financiering van gezondheidsonderzoek. In totaal zullen 129 proefpersonen, die een combinatiebehandeling van chemotherapie met cisplatinum en radiotherapie krijgen, nodig zijn.

De medisch-ethische toetsingscommissie Utrecht heeft dit onderzoek goedgekeurd.

2. Wat is het doel van het onderzoek?

In dit onderzoek bekijken we of patiënten met een lage skeletspiermassa een wekelijks schema cisplatinum beter volhouden dan het standaard (driewekelijkse) schema. Een lage skeletspiermassa betekent dat de omvang van spieren in het lichaam, die nodig zijn voor beweging, kleiner is. Daarnaast bekijken we in dit onderzoek of bijwerkingen hierdoor minder zijn en of minder terugkeer van de ziekte optreedt, de overleving verbetert, een betere kwaliteit van leven verkregen kan worden, en kosten kunnen worden bespaard.

3. Wat is de achtergrond van het onderzoek?

Drie kuren van een hoge dosis cisplatinum (chemotherapie) tijdens zeven weken bestraling (driewekelijks schema) is de standaard behandeling voor hoofd-halskankerpatiënten in het UMC Utrecht. Soms kan ook een wekelijks schema cisplatinum worden gegeven als behandeling. Dit wordt in verschillende andere ziekenhuizen in Nederland ook toegepast als standaard behandeling. Met name bij mensen met een lage skeletspiermassa kan cisplatinum veel bijwerkingen geven. Dan kunnen niet alle kuren volgens het schema gegeven worden. Het effect van de behandeling kan dan minder zijn. Op dit moment kan nog niet goed voorspeld worden bij wie dit zal gebeuren en welke patiënten beter een ander schema kunnen krijgen. Misschien kunnen mensen met een lage skeletspiermassa, een schema van 7 wekelijkse kortere kuren met een lagere dosis beter verdragen dan 3 langere kuren elke drie weken met een hoge dosis. Hierdoor kunnen zij wellicht makkelijker de aanbevolen totaal dosering halen. Het totale effect van de behandeling kan dan beter worden. Door dit onderzoeken uit te voeren, hopen wij een duidelijker beeld te krijgen welk behandelschema voor patiënten met een lage skeletspiermassa beter is.

4. Hoe verloopt het onderzoek?

Hoelang duurt het onderzoek?
Doet u mee met het onderzoek? Dan duurt dat in totaal ongeveer 24 maanden.

Stap 1: bent u geschikt om mee te doen?
Uw skeletspiermassa, lengte, gewicht en voedingsstatus zijn door uw behandelaar bepaald. **Uw skeletspiermassa is laag** waardoor u geschikt bent voor dit onderzoek.

Stap 2: de behandeling
Indien u toestemming geeft voor dit onderzoek, zal loting bepalen welke behandeling u krijgt. De combinatie behandeling van chemotherapie en radiotherapie duurt in totaal 7 weken.

Stap 3: onderzoeken en metingen

Voor dit onderzoek is het niet nodig extra naar het ziekenhuis te komen. De radiotherapie wordt dagelijks gedurende ongeveer 7 weken gegeven. De chemotherapie wordt in de ene groep 3 keer en in de andere groep 7 keer gegeven op een dag dat er ook radiotherapie is. De meeste gegevens die nodig zijn voor het onderzoek zullen uit uw medisch status gehaald worden. Bijwerkingen zullen worden genoteerd op de gebruikelijke wijze.

Wij sturen u vijf keer vier vragenlijsten. De vragen gaan over de kwaliteit van leven en mogelijkheid tot werken. Het kost u ongeveer 30 minuten om deze in te vullen. De onderzoeker belt u drie maanden na de behandeling. U krijgt dan vragen over uw ervaring met de behandeling. Dit telefoongesprek duurt ongeveer tien minuten.

Stap 4: nacontrole

Het onderzoek is afgerond na het verrichten van de standaard onderzoeken tot 24 maanden na de behandeling. De normale controles die bij uw behandeling horen, zullen hierna natuurlijk blijven plaatsvinden.

Wat is er anders dan bij gewone zorg?

Het onderzoek houdt voor u in dat afhankelijk van loting u één van beide behandelschema's krijgt. De totale duur van de behandeling is gelijk. Wanneer u een lage skeletspiermassa heeft zal u gevraagd worden vijf keer een set van vragenlijsten in te vullen.

5. Welke afspraken maken we met u?

We willen graag dat het onderzoek goed verloopt. Daarom maken we de volgende afspraken met u:

- U doet tijdens dit onderzoek niet ook nog mee aan een ander medisch-wetenschappelijk onderzoek zonder overleg met de onderzoekers.
- U komt naar iedere afspraak.
- U neemt contact op met de onderzoeker in deze situaties:
    - U wilt niet meer meedoen met het onderzoek.
    - Uw telefoonnummer, adres of e-mailadres verandert.

6. Van welke bijwerkingen, nadelige effecten of ongemakken kunt u last krijgen?

De te onderzoeken therapie kan bijwerkingen geven. Hierover heeft u voorlichting gekregen van uw behandelend arts en verpleegkundigen. Deze bijwerkingen komen voor bij beide schema's. Het doel van het wekelijkse schema is om te voorkomen dat mensen met een lage skeletspiermassa ernstige bijwerkingen krijgen. De mogelijke bijwerkingen zijn wel vergelijkbaar van aard. Wanneer mensen moeten stoppen met de behandeling door ernstige bijwerkingen, kan het zijn dat een andere behandeling gestart moet worden. Dit kan invloed hebben op de uiteindelijke genezing.

7. Wat zijn de voordelen en de nadelen als u meedoet aan het onderzoek?

Meedoen aan het onderzoek kan voordelen en nadelen hebben. Hieronder zetten we ze op een rij. Denk hier goed over na, en praat erover met anderen.

Omdat u een lage skeletspiermassa hebt, is het misschien voordelig deel te nemen aan dit onderzoek:

- Een voordeel van deelname in dit onderzoek zou kunnen zijn dat u de behandeling met wekelijkse toediening beter verdraagt. Dit is echter nog niet bewezen. Als u de behandeling beter verdraagt dan heeft u minder last van bijwerkingen en is het makkelijker om de behandeling te voltooien.

Meedoen aan het onderzoek kan deze nadelen hebben:

- Meedoen aan het onderzoek kost u extra tijd. U wordt in totaal vijf keer gevraagd vragenlijsten in te vullen. De vragenlijsten kunnen confronterend zijn.
- U krijgt mogelijk 7 keer in dagbehandeling uw chemotherapie in plaats van drie keer tijdens een klinische opname. Dit hangt af van de groep waarin u ingedeeld wordt als u meedoet.
- Het is op dit moment nog onduidelijk welk chemotherapieschema daadwerkelijk het beste is voor mensen met een lage skeletspiermassa.

Wilt u niet meedoen?

U beslist zelf of u meedoet aan het onderzoek. Wilt u niet meedoen? Dan krijgt u de gebruikelijke behandeling voor hoofd-halskanker, namelijk het driewekelijkse schema.

8. Wanneer stopt het onderzoek?

De onderzoeker laat het u weten als er nieuwe informatie over het onderzoek komt die belangrijk voor u is. De onderzoeker vraagt u daarna of u blijft meedoen.

In deze situaties stopt voor u het onderzoek:

- Alle onderzoeken volgens het schema zijn voorbij.
- Twee jaar na de chemotherapie.
- U wilt zelf stoppen met het onderzoek. Dat mag op ieder moment. Meld dit dan meteen bij de onderzoeker. U hoeft er niet bij te vertellen waarom u stopt.
- De behandelend arts vindt het beter voor u om te stoppen.
- Een van de volgende instanties besluit dat het onderzoek moet stoppen:
    - het UMC Utrecht;
    - de overheid, of;
    - de medisch-ethische commissie die het onderzoek beoordeelt.

Wat gebeurt er als u stopt met het onderzoek?

De onderzoekers gebruiken de gegevens die tot het moment van stoppen zijn verzameld. Als u wilt, kunnen verzamelde gegevens worden vernietigd. Geef dit door aan de onderzoeker.

9. Wat gebeurt er na het onderzoek?

Krijgt u de resultaten van het onderzoek?
Ongeveer 48 maanden na de start van dit onderzoek zullen de uitkomsten gepubliceerd worden in de nieuwsbrief van Patiëntenvereniging HOOFD-HALS.

10. Wat doen we met uw gegevens?

Doet u mee met het onderzoek? Dan geeft u ook toestemming om uw gegevens te verzamelen, gebruiken en bewaren.

Welke gegevens bewaren we?
We bewaren deze gegevens:
- uw naam;
- uw geslacht;
- uw adres;
- uw geboortedatum;
- gegevens over uw gezondheid;
- gegevens over uw gebruik van medische voorzieningen;
- (medische) gegevens die we tijdens het onderzoek verzamelen.

Waarom verzamelen, gebruiken en bewaren we uw gegevens?
We verzamelen, gebruiken en bewaren uw gegevens om de vragen van dit onderzoek te kunnen beantwoorden. En om de resultaten te kunnen publiceren.

Hoe beschermen we uw privacy?
Om uw privacy te beschermen geven wij uw gegevens een code. Op al uw gegevens zetten we alleen deze code. De sleutel van de code bewaren we op een beveiligde plek in het UMC Utrecht. Als we uw gegevens verwerken, gebruiken we steeds alleen die code. Ook in rapporten en publicaties over het onderzoek kan niemand terughalen dat het over u ging.

Wie kunnen uw gegevens zien?
Sommige personen kunnen wel uw naam en andere persoonlijke gegevens zonder code inzien. Dit zijn mensen die controleren of de onderzoekers het onderzoek goed en betrouwbaar uitvoeren. Deze personen kunnen bij uw gegevens komen:

- Het onderzoeksteam binnen het UMC Utrecht en, indien van toepassing, uw eigen ziekenhuis.
- Leden van de commissie die de veiligheid van het onderzoek in de gaten houdt.
- Een controleur die door de het UMC Utrecht is ingehuurd.
- Nationale toezichthoudende autoriteiten. Bijvoorbeeld de Inspectie Gezondheidszorg en Jeugd.

Deze personen houden uw gegevens geheim. Wij vragen u voor deze inzage toestemming te geven.

Hoelang bewaren we uw gegevens?

We bewaren uw gegevens 15 jaar in het ziekenhuis en bij de opdrachtgever (het UMC Utrecht) na het einde van het onderzoek.

Mogen we uw gegevens gebruiken voor ander onderzoek?

Uw gegevens kunnen na afloop van dit onderzoek ook nog van belang zijn voor ander wetenschappelijk onderzoek op het gebied van uw aandoening en de behandelmethode. Daarvoor zullen uw gegevens 15 jaar worden bewaard in het ziekenhuis. In het toestemmingformulier geeft u aan of u dit goed vindt. Geeft u geen toestemming? Dan kunt u nog steeds meedoen met dit onderzoek. U krijgt dezelfde zorg.

Wat gebeurt er bij onverwachte ontdekkingen?

Tijdens het onderzoek kunnen we toevallig iets vinden dat belangrijk is voor uw gezondheid of voor de gezondheid van uw familieleden. De onderzoeker neemt dan contact op met uw specialist. U bespreekt dan met uw huisarts of specialist wat er moet gebeuren. U geeft met het formulier toestemming voor het informeren van uw huisarts of specialist.

Kunt u uw toestemming voor het gebruik van uw gegevens weer intrekken?

U kunt uw toestemming voor het gebruik van uw gegevens op ieder moment intrekken. Dit geldt voor het gebruik in dit onderzoek en voor het gebruik in ander onderzoek. Maar let op: trekt u uw toestemming in, en hebben onderzoekers dan al gegevens verzameld voor een onderzoek? Dan mogen zij deze gegevens nog wel gebruiken.

Wilt u meer weten over uw privacy?

- Wilt u meer weten over uw rechten bij de verwerking van persoonsgegevens? Kijk dan op www.autoriteitpersoonsgegevens.nl.
- Heeft u vragen over uw rechten? Of heeft u een klacht over de verwerking van uw persoonsgegevens? Neem dan contact op met degene die verantwoordelijk is voor de verwerking van uw persoonsgegevens. Voor uw onderzoek is dat:
    - Het UMC Utrecht. Zie bijlage A voor contactgegevens, en website.
- Als u klachten heeft over de verwerking van uw persoonsgegevens, raden we u aan om deze eerst te bespreken met het onderzoeksteam. U kunt ook naar de Functionaris Gegevensbescherming van het UMC Utrecht gaan. Of u dient een klacht in bij de Autoriteit Persoonsgegevens.

Waar vindt u meer informatie over het onderzoek?

Op de volgende website vindt u meer informatie over het onderzoek: www.clinicaltrialsregister.eu. Na het onderzoek kan de website een samenvatting van de resultaten van dit onderzoek tonen. U vindt het onderzoek door te zoeken op NL76533.041.21.

11. Krijgt u een vergoeding als u meedoet aan het onderzoek?

De extra testen (vragenlijsten) voor het onderzoek kosten u niets. U krijgt ook geen vergoeding als u meedoet aan dit onderzoek. Indien u onverhoopt voor een extra bezoek aan het ziekenhuis, verband houdende met uw medewerking aan dit onderzoek, reiskosten moet maken zullen wij deze vergoeden.

12. Bent u verzekerd tijdens het onderzoek?

Voor iedereen die meedoet aan dit onderzoek is een verzekering afgesloten. De verzekering betaalt voor schade door het onderzoek. Maar niet voor alle schade. In **bijlage B** vindt u meer informatie over de verzekering en de uitzonderingen. Daar staat ook aan wie u schade kunt melden.

13. We informeren uw huisarts of behandelend specialist

De onderzoeker stuurt uw huisarts of behandelend specialist een bericht om te laten weten dat u meedoet aan het onderzoek. Dit is voor uw eigen veiligheid. Wij vragen ook uw medische voorgeschiedenis en huidige ziektebeloop op bij uw huisarts of behandelend specialist, wanneer dit nodig is voor deze studie. U moet hiervoor toestemming geven op het toestemmingsformulier. Als u geen toestemming geeft, kunt u niet meedoen aan het onderzoek

14. Heeft u vragen?

Vragen over het onderzoek kunt u stellen aan de onderzoeker. Wilt u advies van iemand die er geen belang bij heeft? U kunt de onafhankelijk deskundige, die aan het eind van deze brief genoemd wordt, om aanvullende informatie vragen. Hij/zij weet veel over het onderzoek, maar werkt niet mee aan dit onderzoek.
Heeft u een klacht? Bespreek dit dan met de onderzoeker of de arts die u behandelt. Wilt u dit liever niet? Ga dan naar klachtencommissie van uw ziekenhuis. In bijlage A staat waar u die kunt vinden.

15. Hoe geeft u toestemming voor het onderzoek?

U kunt eerst rustig nadenken over dit onderzoek. Daarna vertelt u de onderzoeker of u de informatie begrijpt en of u wel of niet wilt meedoen. Wilt u meedoen? Dan vult u het toestemmingsformulier in dat u bij deze informatiebrief vindt. U en de onderzoeker krijgen allebei een getekende versie van deze toestemmingsverklaring.

Dank voor uw tijd.

16. Bijlagen bij deze informatie

A. Contactgegevens UMC Utrecht
B. Toestemmingsformulier(en)

Bijlage A: contactgegevens voor het UMC Utrecht

Contactpersoon en uitvoerend onderzoeker:
Drs. A.W.M.A. Schaeffers
Arts-onderzoeker Hoofd-Hals Chirurgische Oncologie
UMC Utrecht Cancer Center, Hoofd-Hals Chirurgische Oncologie
a.w.m.a.schaeffers-2@umcutrecht.nl
088-7550819

Hoofdonderzoeker:
Prof. dr. R. de Bree
Hoofd-hals chirurgisch oncoloog, afdelingshoofd
UMC Utrecht Cancer Center, Hoofd-Hals Chirurgische Oncologie
R.deBree@umcutrecht.nl
088-7550819

Onafhankelijk deskundige:
Dr. P.A.H. Doornaert
UMC Utrecht Cancer Center , Radiotherapie
P.A.H.Doornaert@umcutrecht.nl
088- 7567897
Bereikbaar op werkdagen van 08:30 – 17:00 uur

Klachten: Als u klachten heeft kunt u dit melden aan de arts-onderzoeker of aan uw behandelend arts. Mocht u ontevreden zijn over de gang van zaken bij het onderzoek en een klacht willen indienen dan kunt u contact opnemen met de klachtenbemiddelaars. Deze zijn bereikbaar via tel. 088-755 62 08. Of digitaal via: http://www.umcutrecht.nl/nl/Ziekenhuis/Ervaringen-van-patienten/Een-klacht-indienen.

Functionaris voor de Gegevensbescherming van de instelling:
Gegevensbeschermingprivacy@umcutrecht.nl

Voor meer informatie over uw rechten: www.autoriteitpersoonsgegevens.nl.

Bijlage B: informatie over de verzekering

Het UMC Utrecht heeft een verzekering afgesloten voor iedereen die meedoet aan het onderzoek. De verzekering betaalt de schade die u heeft doordat u aan het onderzoek meedeed. Het gaat om schade die u krijgt tijdens het onderzoek, of binnen 4 jaar na het onderzoek. U moet schade binnen 4 jaar melden bij de verzekeraar.

Bij schade kunt u direct contact leggen met de verzekeraar.

De verzekeraar van het onderzoek is:	
Naam:	CNA Insurance Company (Europe) S.A.
Adres:	Polarisavenue 140
Telefoonnummer:	+31 (0)23 303 6004
E-mail:	Esther.Vanherk@cnahardy.com
Polisnummer:	10201366
Contactpersoon:	Mw. Esther van Herk

De verzekering betaalt maximaal €650.000 per persoon en €5.000.000 voor het hele onderzoek en maximaal 7.500.000 per jaar voor alle onderzoeken van dezelfde opdrachtgever.

Let op: de verzekering dekt de volgende schade **niet**:

- Schade door een risico waarover we u informatie hebben gegeven in deze brief. Maar dit geldt niet als het risico groter bleek te zijn dan we van tevoren dachten. Of als het risico heel onwaarschijnlijk was.
- Schade aan uw gezondheid die ook zou zijn ontstaan als u niet aan het onderzoek had meegedaan.
- Schade die ontstaat doordat u aanwijzingen of instructies niet of niet goed opvolgde.
- Schade aan de gezondheid van uw kinderen of kleinkinderen.
- Schade door een behandelmethode die al bestaat. Of door onderzoek naar een behandelmethode die al bestaat.

Deze bepalingen staan in het 'Besluit verplichte verzekering bij medisch-wetenschappelijk onderzoek met mensen 2015'. Dit besluit staat in de Wettenbank van de overheid (https://wetten.overheid.nl).

Bijlage C: toestemmingsformulier proefpersoon

Behorende bij:
Onderzoek naar het volbrengen van de geplande kuren cisplatinum bij hoofd-halskankerpatiënten met een lage skeletspiermassa door verschillende toedieningsschema's.

- Ik heb de informatiebrief gelezen. Ook kon ik vragen stellen. Mijn vragen zijn voldoende beantwoord. Ik had genoeg tijd om te beslissen of ik meedoe.
- Ik weet dat meedoen vrijwillig is. Ook weet ik dat ik op ieder moment kan beslissen om toch niet mee te doen met het onderzoek. Of om ermee te stoppen. Ik hoef dan niet te zeggen waarom ik wil stoppen.
- Ik geef de onderzoeker toestemming om mijn huisarts en behandelend specialist te laten weten dat ik meedoe aan dit onderzoek.
- Ik toestemming dat data dat van belang is voor het onderzoek naar het onderzoeksteam in het UMC Utrecht wordt gestuurd.
- Ik geef de onderzoeker toestemming om informatie op te vragen bij mijn huisarts en behandelend specialist over mijn voorgeschiedenis en huidige ziekte.
- Ik weet dat voor de controle van het onderzoek sommige mensen al mijn gegevens kunnen inzien. Die mensen staan in deze informatiebrief. Ik geef deze mensen toestemming om mijn gegevens in te zien voor deze controle.
- Ik geef toestemming om mijn gegevens op de onderzoekslocatie nog 15 jaar na dit onderzoek te bewaren.
- Wilt u in de tabel hieronder ja of nee aankruisen?

Ik geef toestemming om, in het geval ik tijdens de looptijd van het onderzoek overlijd, mijn officiële doodsoorzaakgegevens op te vragen bij het Centraal Bureau voor de Statistiek	Ja ☐	Nee☐
Ik geef toestemming om mijn gegevens te bewaren om dit te gebruiken voor ander onderzoek, zoals in de informatiebrief staat.	Ja ☐	Nee☐
Ik geef toestemming om mij eventueel na dit onderzoek te vragen of ik wil meedoen met een vervolgonderzoek.	Ja ☐	Nee☐

- Ik wil meedoen aan dit onderzoek.

Mijn naam is (proefpersoon): ………………………………..
Handtekening: ……………………… Datum : __ / __ / __

Ik verklaar dat ik deze proefpersoon volledig heb geïnformeerd over het genoemde onderzoek.

Wordt er tijdens het onderzoek informatie bekend die die de toestemming van de proefpersoon kan beïnvloeden? Dan laat ik dit op tijd weten aan deze proefpersoon.

Naam onderzoeker (of diens vertegenwoordiger):……………………………….
Handtekening:……………………… Datum: __ / __ / __

(indien van toepassing)
Aanvullende informatie is gegeven door:
Naam:………………………………..
Functie:………………………………
Handtekening:……………………… Datum: __ / __ / __

De proefpersoon krijgt een volledige informatiebrief mee, samen met een getekende versie van het toestemmingsformulier.
