# Supplementary material for: Low dose cisplatin weekly versus high dose cisplatin every three weeks in primary chemoradiotherapy in head and neck cancer patients with low skeletal muscle mass: The CISLOW-study protocol
Source: PLoS One. 2023 Nov 27;18(11):e0294147. doi: 10.1371/journal.pone.0294147 (PMC10681175; doi:10.1371/journal.pone.0294147)
Supplement: S2 File — (PDF) [file pone.0294147.s004.pdf]

Low dose cisplatin weekly versus high dose cisplatin every three weeks in primary chemoradiation in sarcopenic head and neck cancer patients

1. General features

1.1. Please fill in the table below. When not applicable (yet), please fill in N/A.

DMP template version	29 (don't change)
ABR number *(only for human-related research)*	To be determined
METC number *(only for human-related research)*	To be determined
DEC number *(only for animal-related research)*	N/A
Acronym/short study title	CISLOW
Name Research Folder	To be determined
Name Division	Division Cancer Center
Name Department	Department of Head and Neck Surgical Oncology
Partner Organization	
Start date study	01-01-2021
Planned end date study	29-12-2025
Name of datamanager consulted*	Jacco van der Laan
Check date by datamanager	26-01-2021

1.2 Select the specifics that are applicable for your research.

- Use of Questionnaires
- Prospective study
- Multicenter study
- Interventional study
- WMO

2. Data Collection

2.1 Give a short description of the research data.

Subjects	Volume	Data Source	Data Capture Tool	File Type	Format	Storage space
Human	129	Various (e.g. HIX)	Castor	Quantitative	.csv	0-10 GB
Human	129	eCRF	Castor	Quantitative	various (e.g. .csv)	0-10 GB
Human	129	Computed Tomography scans (CT scans), MRI scans and PET CT scans	Research Imaging Architecture	Imaging	.dcm	101-1000 GB
Human	129	Questionnaires	Castor	Quantitative	.csv	0-10 GB

2.2 Do you reuse existing data?

- Yes, please specify

In this study we use data from EPD (HiX) which is collected for clinical care.

2.3 Describe who will have access to which data during your study.

Type of data	Who has access
Personal data (EPD Hix and PACs radiology)	PI, study team, data manager
Key table linking study specific IDs to Patient IDs	PI, executive researcher, data manager
Pseudonymized data (Castor and RIA)	PI, study team, data manager

2.4 Describe how you will take care of good data quality.

To ensure data quality, completeness and integrity, a GCP-compliant database and certified Data Capture Tool(Castor) will be used based on data capture with data trail. Data quality will be checked by a local monitor from the UMCU since this study poses negligible risk. Data collection will be frozen before analysis.

Patient digital imaging data will be stored for study purposes at the Research Imaging Archive (RIA) facility of the imaging division of the UMC Utrecht. Hospitals may transfer digital data into the RIA through secure connections. The RIA shields patient identifiable information through pseudonymized identifiers (i.e., study number) and only allows access to authorized researchers.

Data will be matched by study subject code.

#	Question	Yes	No	N/A
1.	Do you use a certified Data Capture Tool or Electronic Lab Notebook?	x		
2.	Have you built in skips and validation checks?	x		
3.	Do you perform repeated measurements?	x		
4.	Are your devices calibrated?			x
5.	Are your data (partially) checked by others (4 eyes principle)?			x
6.	Are your data fully up to date?	x		
7.	Do you lock your raw data (frozen dataset)	x		
8.	Do you keep a logging (audit trail) of all changes?	x		
9.	Do you have a policy for handling missing data?	x		
10.	Do you have a policy for handling outliers?	x		

2.5 Specify data management costs and how you plan to cover these costs.

#	Type of costs	Division ("overhead")	Funder	Other (specify)
1.	Data capturing tool Castor	x		
2.	Datamanagement	x		
3.	Monitoring		x	
4.	Questionnaire license fee		x	
5.	SliceOmatic license		x	
6.	METC		x	
7.	Open access		x	
8.	Storage	x		
9.	Archiving			x

1. License fee for using Castor

9. Where data will be archived and how these costs will be covered has yet to be determined. This answer will be updated later.

2.6 State how ownership of the data and intellectual property rights (IPR) to the data will be managed, and which agreements will be or are made.

Our data collection consists of data from questionnaires and medical records that have been collected among 129 head and neck patients who will undergo chemoradiation. Since this is a multicenter study, other centra must also be able to enter the database. Only the principal investigators and executive researcher will have access to the source data. The key to the code will be safeguarded by the study coordinators. Data will be kept 15 years.

The data is collected in a relatively large patient group and is very valuable for further, broader studies in the world. Therefor we aim at publishing results in an international journal. I will not be possible to track back these results to the individual patient.

3. Personal data (Data Protection Impact Assessment (DPIA) light)

Will you be using personal data (direct or indirect identifying) from the Electronic Patient Dossier (EPD), DNA, body material, images or any other form of personal data?

- Yes, go to next question

3.1 Describe which personal data you are collecting and why you need them.

Which personal data?	Why?
Name, adress, email, telephone number	Participition in the research. Data wil be encrypted.
Images (CT/MRI/PET-CT)	To determine whether patients have low skeletal muscle mass. This is necessary to determine if patients should be randomized in this low skeletal muscle mass group. Research can not be performed without the data.
Treatment plan, scores (ECOG performance status, ACE-27, Charlson Comorbidity Index, TNM classification), laboratory results, gender, age, weight, stature (length), smoking, history, use of alcohol, loss of weight, toxicities.	These characteristics and potential predictive factors for cispatin related toxicity are neccessary for research and outcomes.

3.2 What legal right do you have to process personal data?

- Study-specific informed consent

Sharing of any patient data with other parties, long term preservation of data or re-use of data will only be possible if patients have signed informed consent. These questions are included in the informed consent form. Sharing or re-use of data will only be possible of data that can not be traced back to the individual patient.
To ensure this, UMC Utrecht will remove personal data from the files that will be shared.

3.3 Describe how you manage your data to comply to the rights of study participants.

The data are pseudonymized and the linking table to personal data is saved. An authorized person manages the linking table, can re-identify study participants when necessary and deliver, correct or delete the data.

Right	Example answers
Right of Access	Research data are coded, but can be linked back to personal data, so we can generate a personal record at the moment the person requires that. This needs to be done by an authorized person.
Right of Rectification	The authorized person will give the code for which data have to be rectified.
Right of Objection	We use informed consents.
Right to be Forgotten	In the informed consent we state that the study participant can stop taking part in the research. Removal of collected data from the research database cannot be granted because this would result in a research bias.

3.4 Describe the tools and procedures that you use to ensure that only authorized persons have access to personal data.

We use the secured Research Folder Structure that ensures that only authorized personnel has access to personal data, including the key table that links personal data to the pseudoID.

Also we make use of GCP compliant Electronic Data Capture software (Castor). No personal data other than email address will be used in the EDC, but this is encrypted for the users in such a way that users can send emails to subjects without seeing the actual email address.

Patient digital imaging data for study purposes will be stored at the Research Imaging Archive (RIA) facility of the imaging division of the UMC Utrecht. For safe processing of images, RIA will be used (uses pseudoanomization in order to guarantee safe processing). Only authorized personnel can access the (pseudomized) imaging in the RIA container via personal login. The linkage table for the pseudonymized images will also be stored at the Research Imaging Archive (RIA), the container can only be accessed by users with the proper rights.

Since this will be a multi-center study, hospitals may transfer digital data into the RIA through secure connections. The RIA shields patient identifiable information through pseudonymized identifiers (i.e., study number) and only allows access to authorized researchers

3.5 Describe how you ensure secure transport of personal data and what contracts are in place for doing that.

After inclusion in the study, the PI at the collaborating center needs to fill in the eCRF.
In case we need to transport personal data with colleagues, we use Surffilesender with encryption.

4. Data Storage and Backup

4.1 Describe where you will store your data and documentation during the research.

Paper dossiers will be stored safely (e.g. in a locked room or cabinet or outside UMC Utrecht; a project specific procedure is in place for access to the paper dossiers and the documentation of the procedure is stored in de research folder. The digital files will be stored in the Secured Research Folder (Beveiligde Onderzoeks Map) using RFS.

4.2 Describe your backup strategy or the automated backup strategy of your storage locations.

All (research) data is stored on UMC-Utrecht Network drives, from which twice a day a backup is made automatically by the local IT department of the UMCU. Automatic backups will be made in Castor EDC during data collection. At the end of data collection all data are exported, saved in the research folder and backed up by the UMC Utrecht backup system.

5. Metadata and Documentation

5.1 Describe the metadata that you will collect and which standards you use.

We do not use metadata standards yet. For the data stored in the data capturing tool, The research team prepared a codebook of my research database.
If applicable, the following standards will be used:
CDISC (Clinical Data Interchange Standard Consortium)
ICD-O (International Classification of Diseases for Oncology)
TNM-O (The Union for International Cancer Control)
If applicable, naming conventions of the items will be determined at a later timepoint.

5.2 Describe your version control and file naming standards.

We will distinguish versions of the research project by indicating the version in the filename of the master copy by adding a code after each edit, for example V1.1 dd dd-mo-yr.
(first number for major versions, last for minor versions). The most recent copy at the master location is always used as the source, and before any editing, this file is saved with the new version code in the filename. The file with the highest code number is the most recent version.
It is anticipated to register the project at www.trialregister.nl

6. Data Analysis

6 Describe how you will make the data analysis procedure insightful for peers.

A detailed description will be available in the study protocol (C1) of the METC application. The researcher is going to write a paper and publish it, which will make the research accessible to peers. It won't be possible to trace back data to an individual patient.

7. Data Preservation and Archiving

7.1 Describe which data and documents are needed to reproduce your findings.

In view of the regulation for Clinical Trials, data will be saved for at least 15 years with the goal to be able to go back to patient level. After finishing the project all documents and data are stored at the UMC Utrecht and maintained by a datamanager.

7.2 Describe for how long the data and documents needed for reproducibility will be available.

In view of the regulation for Clinical Trials, we need to store all data for at least 15 years with the goal to be able to go back to patient level.

7.3 Describe which archive or repository (include the link!) you will use for long-term archiving of your data and whether the repository is certified.

After finishing the project, the data package will be stored at the UMC Utrecht Research Folder Structure and is under the responsibility of the Principal Investigator of the research group. When the UMC Utrecht repository is available, the data package will be published here.

7.4 Give the Persistent Identifier (PID) that you will use as a permanent link to your published dataset.

I will be using a DOI-code and will update this plan as soon as I have the code.

8. Data Sharing Statement

8.1 Describe what reuse of your research data you intend or foresee, and what audience will be interested in your data.

Our data will possibly be shared with third parties after approval of the principle investigator and approval of the UMC Utrecht Data Access Committee, criteria will be determined later.
Our data can be of interest for further research. We will make sure it won't be possible to trace back to the individual patient, using a code which won't be published.

8.2 Are there any reasons to make part of the data NOT publicly available or to restrict access to the data once made publicly available?

- Yes (please specify)

Our data will be shared with third parties after approval of the Principle Investigator. The criteria and time period will be determined on a case-by-case basis.

8.3 Describe which metadata will be available with the data and what methods or software tools are needed to reuse the data.

The publication will have open access. The study protocol and this Data Management Plan will also be available.

8.4 Describe when and for how long the (meta)data will be available for reuse

- Other (please specify)

Our data will possibly be shared with third parties after approval of the principle investigator and approval of the UMC Utrecht Data Access Committee, criteria will be determined later.

8.5 Describe where you will make your data findable and available to others.

Our data will possibly be shared with third parties after approval of the principle investigator and approval of the UMC Utrecht Data Access Committee, criteria will be determined later.
