# Supplementary material for: Low dose cisplatin weekly versus high dose cisplatin every three weeks in primary chemoradiotherapy in head and neck cancer patients with low skeletal muscle mass: The CISLOW-study protocol
Source: PLoS One. 2023 Nov 27;18(11):e0294147. doi: 10.1371/journal.pone.0294147 (PMC10681175; doi:10.1371/journal.pone.0294147)
Supplement: S3 File — (PDF) [file pone.0294147.s005.pdf]

Template Monitorplan –matig risico- WMO-plichtig onderzoek

<u>Invulinstructie:</u>

- Om de velden in te kunnen vullen, is het nodig om in de gele balk bovenaan het document 'Document bewerken' in te schakelen.
- Elk onderdeel dient ingevuld te worden.
- Uitleg voor de verschillende onderwerpen verkrijgt u door de bijbehorende bladwijzer te lezen. Dit doet u door *Ctrl+klik* op het betreffende onderstreepte onderwerp.
- De cursieve tekst dient vervangen te worden door onderzoeks-specifieke informatie.
- Het uiteindelijke monitorplan bestaat alleen uit de tabellen op pagina1 en 2. De overige pagina's dienen alleen als achtergrondinformatie en bevatten daarom geen voettekst en paginanummering.
- Op de laatste pagina bevindt zich een afkortingenlijst.
- Het maken van een eigen voettekst met daarin versienummer, -datum en titel kan via de instructie hier onderaan de pagina.

<u>Uitprinten:</u>

- Controleer na uitprinten of alle velden zijn ingevuld.
- Creëer voor uw eigen administratie een PDF-file van het monitorplan (Selecteer printer -> PDF Creator).

<u>Een wijziging in het onderzoeksprotocol kan een wijziging van het monitorplan tot gevolg hebben.</u>

In het kader hieronder tekst voor voettekst invoeren:

Cislow study NL76533.041.21 versie 2.0

Neem bij vragen contact op met de kwaliteitscoördinator van de eigen divisie.

Template Monitorplan –matig risico- WMO-plichtig onderzoek

Monitorplan

**1. ALGEMENE GEGEVENS**	
Titel studie	Low dose cisplatin weekly versus high dose cisplatin every three weeks with primary radiotherapy in sarcopenic head and neck cancer patients
METC-nummer	Klik hier als u tekst wilt invoeren.
ABR-nummer	NL76533.041.21
Deelnemende centra + hoofdonderzoeker	Universitair Medisch Centrum Utrecht, prof. dr. R. de Bree<br>Antoni van Leeuwenhoek, dr. J.P. de Boer<br>Amsterdam Universitair Medische Centra, locatie VUmc, dr. J. Voortman
(Centrale) hoofdonderzoeker	Prof. dr. R. de Bree
Verrichter	Universitair Medisch Centrum Utrecht
Risicoclassificatie	Matig = matig intensieve monitoring
Aantal patiënten + looptijd onderzoek	129 patienten gedurende 48 maanden
Monitor	Aangesteld via Julius Centrum

**2. MONITORING: FREQUENTIE**	
Initiatie visite	Per centrum 1x, gelijktijdig middels een centrale kick-off
Monitor visite	Per centrum jaarlijks minimaal 2 visites (waarvan jaarlijks in ieder geval 1 on-site[1] visite per centrum); afhankelijk van de inclusie snelheid, duur van het onderzoek, aantal onderzoeksdeelnemers en eerder geobserveerde deviaties.
Close-out visite	Per centrum 1 x welke remote wordt uitgevoerd en gecombineerd wordt met de laatste monitorvisite.

**3. MONITORING: INHOUD**	
Inclusiestroom	Controle van de inclusiesnelheid en het uitvalspercentage.
Study File	Controle aanwezigheid en volledigheid. *Beschrijf welke Study File(s) van toepassing is/zijn.*
Informed Consent	Aanwezigheid: minimaal 50% (indien mogelijk 100%) van het totaal aantal geïncludeerde onderzoeksdeelnemers[2] per centrum, indien mogelijk.<br>Navragen van het proces en verificatie op uitvoer van het volledige IC proces bij minimaal 25% van het totaal aantal geïncludeerde onderzoeksdeelnemers[2] per centrum, indien mogelijk.
In- en exclusie criteria	Verificatie van minimaal 25% van het totaal aantal geïncludeerde onderzoeksdeelnemers[2] per centrum.
Source Data Review en Source Data Verification	Verificatie van minimaal 25% van het totaal aantal geïncludeerde onderzoeksdeelnemers[2] per centrum (SDV en SDR). Verificatie op basis van een tevoren gedefinieerde lijst van variabelen, inclusief

[1] Alternatief van on-site monitoring is Centralized monitoring. Dit is Remote monitoring (waarbij de monitor op afstand vragen stelt of documenten opvraagt, in geen geval documenten met persoonsgegevens) of Statistical monitoring (waarbij verzamelde data samen met een statisticus wordt bekeken)
[2] Geïncludeerde onderzoeksdeelnemers = getekend informed consent.

	primair eindpunt, die in duidelijke relatie staan tot de veiligheid en geldigheid van het onderzoek.
Serious Adverse Events (SAEs/SADEs/SUSARs/USADEs)	De onderzoeksdeelnemers bij wie de steekproef voor de SDV/SDR wordt uitgevoerd worden ook gecontroleerd op niet gerapporteerde SAEs.<br>Daarnaast controle van 25% van de gerapporteerde SAEs/SADEs/SUSARs/USADEs.
Onderzoeksproduct	Controleer product accountability van onderzoeksdeelnemers die geselecteerd zijn voor de SDV en welke instructies onderzoeksdeelnemers mee krijgen.
Onderzoeksprocedures	Controleer of instructies voor uitvoer van onderzoeksprocedures aanwezig zijn en of onderzoekspersoneel getraind is in het uitvoeren van onderzoeksprocedures.
Onderzoeksdata	Controleer of onderzoeksdata verzameld wordt in een gevalideerde database.
Apparatuur	Verifieer of gebruikte apparatuur mits deze een functie hebben bij het bepalen van het primair eindpunt opgenomen zijn in het kwaliteitsborgingssysteem/programma.
Lab & Apotheek	*Indien van toepassing zie bladwijzer.*
Aanvullende opmerkingen	Klik hier als u tekst wilt invoeren.

4. MONITORING: VERSLAGLEGGING

De monitor zal een schriftelijk verslag (rapport) maken na ieder bezoek aan een onderzoekslocatie (centrum). Het monitorvisite rapport bevat:

- Een samenvatting van hetgeen de monitor heeft beoordeeld
- Een algemene beschrijving van de kwaliteit
- Een opsomming van belangrijke bevindingen/feiten, afwijkingen en tekortkomingen
- Een overzicht met te nemen maatregelen en aanbevelingen om naleving van het onderzoeksprotocol te garanderen
- De "overall" conclusie

De lokale hoofdonderzoeker ontvangt na alle mogelijke vormen van monitoring van het eigen centrum de begeleidende brief en de 'Monitoring Issues and Actions Tracker' en dient deze te bewaren in de Investigator Site File.
De verrichter*, indien het UMC Utrecht, ontvangt na alle mogelijke vormen van monitoring van het eigen of een deelnemend centrum het originele rapport en een origineel (van eigen centrum) of kopie (van deelnemend centrum) van de begeleidende brief en de 'Monitoring Issues and Action Tracker'. Deze dienen bewaard te worden in de Monocenter Study File (bij monocenter onderzoek), Investigator Site File of Center Master File.

Deze verslaglegging moet door de centrale hoofdonderzoeker en de lokale hoofdonderzoeker bewaard worden en bij een audit ter inzage beschikbaar zijn.

De centrale hoofonderzoeker van de verrichter* (origineel) en de onderzoekslocatie (kopie) ontvangen ook het (centrale) initiatie visite rapport en het (centrale) close-out visite rapport. Indien van toepassing zullen ook overige relevante contacten betreffende het onderzoek schriftelijk vastgelegd worden.
Van alle rapportages zal een kopie naar de kwaliteitscoördinator van de divisie waar het onderzoek wordt opgezet/uitgevoerd worden gestuurd.

* Bij onderzoek met een externe verrichter ontvangt de centrale hoofdonderzoeker kopieën in plaats van originele documenten. De lokale hoofdonderzoeker in het UMC Utrecht ontvangt dan de originele documenten.

Risicoclassificatie:
Het risico, op basis van de NFU risicoclassificatie, wordt vastgesteld door de (centrale) hoofdonderzoeker. Zie NFU Richtlijn Kwaliteitsborging mensgebonden onderzoek, hoofdstuk 4. De METC geeft een uiteindelijk oordeel over de risicoclassificatie.
De verschillende classificaties kennen ieder hun eigen eisen wat betreft minimale monitoring.

Monitor:
Geef aan welke gekwalificeerde, externe monitor wordt ingezet.

Frequentie van de monitoring (per deelnemend centrum):
Per centrum jaarlijks minimaal 2 visites (waarvan jaarlijks in ieder geval 1 on-site[2] visite per centrum); afhankelijk van de inclusie snelheid, duur van het onderzoek, aantal onderzoeksdeelnemers en eerder geobserveerde
deviaties.
Daarnaast zal er voorafgaand aan het onderzoek een "initiatievisite" en aan het einde van het onderzoek een "close-out visite" gepland worden.
Een initiatievisite zal niet langer dan een dagdeel in beslag nemen en wordt uitgevoerd wanneer de goedkeuringsbrief van de METC binnen is. Tijdens de initiatievisite wordt de lijst "Checklist Essential Start-up Documents" doorgenomen. Pas wanneer de essentiële documenten die noodzakelijk zijn om een studie te mogen starten aanwezig zijn, kan er gestart worden met includeren.
Het is mogelijk om de initiatievisite te vervangen door een (centrale) kickoffmeeting. In dat geval dient hiervan een rapport of notulen aanwezig te zijn in de study file.

Het is mogelijk om de laatste monitorvisite te combineren met de close-out visite.
Ook is het mogelijk om een *remote close out* visite uit te voeren. Dit kan door middel van een checklijst die gestuurd wordt aan de onderzoekslocatie waarop de onderzoeker van de onderzoekslocatie tekent ter bevestiging.

Voor een inschatting van het benodigde aantal uren per monitorvisite, kunt u gebruik maken van het rekenmodel monitorkosten.

Study Files:
Tijdens een monitor visite zal controle op aanwezigheid en volledigheid plaatsvinden van de volgende study files:
- Monocenter Study: een Monocenter Study File
- Multicenter Study: een Study Master File ("overkoepelende map" bij de verrichter), Center Master File ("schaduwmap" van deelnemend centrum bij verrichter), Investigator Site File ("werkmap" bij deelnemend centrum)

Informed Consent:
1 - Controle op aanwezigheid van minimaal 50% (indien mogelijk 100%) van het totaal aantal geïncludeerde onderzoeksdeelnemers[2] per centrum, indien mogelijk.

2 - Navragen informed consent proces en verificatie van het volledige IC proces van minimaal 25% van het totaal aantal geïncludeerde onderzoeksdeelnemers[2] per centrum, indien mogelijk.

---

[2] Alternatief van on-site monitoring is Centralized monitoring. Dit is Remote monitoring (waarbij de monitor op afstand vragen stelt of documenten opvraagt, in geen geval documenten met persoonsgegevens) of Statistical monitoring (waarbij verzamelde data samen met een statisticus wordt bekeken)
[2] Geïncludeerde onderzoeksdeelnemers = getekend informed consent.

In-/exclusiecriteria:
Verificatie van minimaal 25% van het totaal aantal geïncludeerde onderzoeksdeelnemers per centrum.
Indien fout-geïncludeerde onderzoeksdeelnemers in het onderzoek zijn opgenomen (het schenden van exclusiecriteria in relatie tot veiligheid is hier vooral van belang) wordt de steekproef naar inzicht uitgebreid ongeacht de mate van intensiteit van monitoring.

Source Data Review en Source Data Verification:
Source data verification (SDV) is vergelijking van brongegevens met (e)CRF gegevens. Source data review (SDR) is een beoordeling van brondocumentatie om de kwaliteit van de bron te controleren, compliance van protocollen te controleren en kritieke processen te waarborgen (bron: TransCelerate) en of voor verzamelde data een bron aanwezig is (medische status).

Verificatie van minimaal 25% van het totaal aantal geïncludeerde onderzoeksdeelnemers (SDV en SDR). Verificatie op basis van een tevoren gedefinieerde lijst van variabelen, inclusief primair eindpunt, die in duidelijke relatie staan tot de veiligheid en geldigheid van het onderzoek.

Serious Adverse Events (SAEs/SADEs/SUSARs/USADEs):
De onderzoeksdeelnemers bij wie de steekproef voor de SDV/SDR wordt uitgevoerd worden ook gecontroleerd op niet gerapporteerde SAEs.
Daarnaast controle van 25% van de gerapporteerde SAEs/ SADEs/SUSARs/ USADEs.

Indien de rapportage en/of geëigende melding van ernstige bijwerkingen of ernstig ongewenste voorvallen onvolledig of incorrect is, wordt de steekproef naar inzicht uitgebreid ongeacht de mate van intensiteit van monitoring. Indien deze onregelmatigheden SUSARs of USADEs betreffen dient de steekproef uitgebreid te worden naar 100%.

Meer informatie met betrekking tot de meldingsprocedure: zie de betreffende ‘Ongewenste voorvallen flow’ in iProva.

Onderzoeksproduct:
Controleer product accountability van onderzoeksdeelnemers die geselecteerd zijn voor de SDV en welke instructies onderzoeksdeelnemers mee krijgen.

Product accountability controleren op onderzoeksdeelnemer, afdeling en/of apotheek niveau. (opslag van producten, vervaldatum, binnenkomst aantal apotheek, uitgifte apotheek/uitgifte aan onderzoeksdeelnemer, doseringen, teruggave/vernietiging etc.)

Onderzoeksprocedures:
Bijvoorbeeld: randomisatie, deblindering, datamanagement en privacy.
Controleer of instructies voor uitvoer van onderzoeksprocedures aanwezig zijn en of onderzoekspersoneel getraind is in het uitvoeren van onderzoeksprocedures.

Onderzoeksdata:
Controleer of onderzoeksdata verzameld wordt in een gevalideerde database.

Apparatuur:
Verifieer of gebruikte apparatuur mits deze een functie hebben bij het bepalen van het primair eindpunt opgenomen zijn in het kwaliteitsborgingssysteem/programma.

Laboratorium en apotheek:
Controleer of laboratorium/apotheek gecertificeerd is/zijn voor de taken die ze uitvoeren voor het betreffende onderzoek. Bij geneesmiddelenonderzoek wordt de apotheek één of meer keer

gedurende het onderzoek bezocht. Indoen laboratorium onderdeel is van het primair eindpunt dan verificatie op laboratoriumprocedures (oa opslag, temperatuur en dergelijke).

**Verklaring gebruikte afkortingen**:	
IC	Informed Consent
METC	Medisch Ethische Toetsingscommissie
NFU	Nederlandse Federatie Universitair Medische Centra
SADE	Serious Adverse Device Event (ernstig ongewenst voorval mogelijk gerelateerd aan het medisch hulpmiddel)
SAE	Serious Adverse Event (ernstig ongewenst voorval)
SUSAR	Suspected Unexpected Serious Adverse Reaction (vermoeden van onverwacht ernstige bijwerking)
SOP	Standard Operating Procedure
USADE	Unanticipated Serious Adverse Device Effect
