# Supplementary material for: Low dose cisplatin weekly versus high dose cisplatin every three weeks in primary chemoradiotherapy in head and neck cancer patients with low skeletal muscle mass: The CISLOW-study protocol
Source: PLoS One. 2023 Nov 27;18(11):e0294147. doi: 10.1371/journal.pone.0294147 (PMC10681175; doi:10.1371/journal.pone.0294147)
Supplement: S4 File — (PDF) [file pone.0294147.s006.pdf]

Low dose cisplatin weekly versus high dose cisplatin every three weeks in primary chemoradiation in sarcopenic head and neck cancer patients

CISLOW-study v6.0. dd 20-02-2023

PROTOCOL TITLE 'Low dose cisplatin weekly versus high dose cisplatin every three weeks in primary chemoradiation in sarcopenic head and neck cancer patients'

**Protocol ID**	**NL76533.041.21**
**Short title**	**Low dose cisplatin in sarcopenic head and neck cancer patients**
**EudraCT number**	2021-002634-16
**Version**	**6.0**
**Date**	**20-02-2023**
**Coordinating investigator/project leader**	Prof. dr. R. de Bree, MD, PhD<br>University Medical Center Utrecht Department of Head and Neck Surgical Oncology<br>Huispost Q05.4.300<br>PO Box 85500 3508GA Utrecht<br>Phone: 088-7550819<br>r.debree@umcutrecht.nl
**Principal investigator(s) (in Dutch: hoofdonderzoeker/ uitvoerder**	**University Medical Center Utrecht**<br>Prof. dr. R. de Bree<br>University Medical Center Utrecht Department of Head and Neck Surgical Oncology<br>Huispost Q05.4.300<br>PO Box 85500 3508GA Utrecht<br>Phone: 088-7550819<br>r.debree@umcutrecht.nl<br>**Amsterdam Universitair Medische Centra**<br>Dr. J. Voortman<br>VUmc Cancer Center Amsterdam, Department of Medical Oncology<br>j.voortman@amsterdamumc.nl<br>**NKI/Antoni van Leeuwenhoek**<br>Dr. J.P. De Boer<br>Antoni van Leeuwenhoek hospital, Department of Medical Oncology<br>j.d.boer@nki.nl

	**Noordwest Ziekenhuisgroep**<br>Dr. M.P. Hendriks, Noordwest Ziekenhuisgroep, Department of Medical Oncology<br>m.p.hendriks@nwz.nl<br><br>**Leiden University Medical Center**<br>Dr. M. Slingerland, Leiden University Medical Center Department of Medical Oncology<br>m.slingerland@lumc.nl<br><br>071-526-1990
**Study committee/other investigators**	Dr. L.A. Devriese<br>University Medical Center Utrecht Department of Medical Oncology<br><br>Huispost Q05.4.300<br>PO Box 85500 3508GA Utrecht<br>Phone: 088-755555<br>L.A.Devriese@umcutrecht.nl<br><br>Dr. G.W.J. Frederix<br>Universitair Medisch Centrum Utrecht<br>Postbus 85500<br>3508 GA UTRECHT<br>Phone: 0887555555<br>G.W.J.Frederix@umcutrecht.nl<br><br>Prof. dr. C. van Gils<br>Universitair Medisch Centrum Utrecht<br>Postbus 85500<br>3508 GA UTRECHT<br>Phone: 0887553014<br>C.vanGils@umcutrecht.nl<br><br>Dr. J.W. Dankbaar<br>University Medical Center Utrecht Department of Radiology<br><br>Huispost E01.132<br>Postbus 85500<br>3508 GA UTRECHT<br>Phone: 0887574341<br>J.W.Dankbaar@umcutrecht.nl<br><br>Drs. A.W.M.A. Schaeffers

	University Medical Center Utrecht Department of Head and Neck Surgical Oncology<br>Huispost Q05.4.300<br>PO Box 85500 3508GA Utrecht<br>Phone: 088-67805<br>a.w.m.a.schaeffers-2@umcutrecht.nl<br>Drs. M.A. van Beers (deputy of drs. Schaeffers)<br>University Medical Center Utrecht Department of Head and Neck Surgical Oncology<br>Huispost D05.44<br>PO Box 85500 3508GA Utrecht<br>Phone: 088-69114<br>m.a.vanbeers-3@umcutrecht.nl
**Sponsor (in Dutch: verrichter/opdrachtgever)**	UMC Utrecht
**Subsidising party**	ZonMw
**Independent expert (s)**	Dr. P.A.H. Doornaert<br>Universitair Medisch Centrum Utrecht<br>Postbus 85500<br>3508GA Utrecht<br>P.A.H.Doornaert@umcutrecht.nl<br>Phone: 088755555<br>Dr. N.W.C.J. van de Donk, Amsterdam University Medical Center<br>Dr. A.M. Bergman, Antoni van Leeuwenhoek<br>Dr. M.A. de Jong, radiotherapist Leiden University Medical center<br>Phone: 071 526 9111
**Laboratory sites <*if applicable*>**	*NA*

**Pharmacy *<if applicable>***	***Dr. H. Crommelin,*** *pharmacy University Medical Center Utrecht*<br>*KGO-Apotheek@umcutrecht.nl*<br>*Medication will be according to local hospital guidelines (standard)*<br><br>***Dr. M. Kemper****, ziekenhuis-apotheker, afdelingshoofd,*<br>*onderzoeksmedicatie@vumc.nl*<br><br>***Prof. dr. A.D.R Huitema,*** *apotheker*<br>*Antoni van Leeuwenhoek Apotheek*<br>*Plesmanlaan 121, 1066CX Amsterdam*<br>*Tel.: 020-512 4481*<br>*Fax: 020-512 4449*<br>*a.huitema@nki.nl*<br><br>**P.H.A.M. Kloeg,** apotheker Noordwest Ziekenhuisgroep<br>p.kloeg@nwz.nl

PROTOCOL SIGNATURE SHEET

Name	Signature	Date
**Sponsor or legal representative:**<br>***Prof. dr. H.M. Verkooijen***		
**Principal Investigator:**<br>***Prof. Dr. R. de Bree, MD, PhD***		

TABLE OF CONTENTS

1. INTRODUCTION AND RATIONALE 13
2. OBJECTIVES 16
3. STUDY DESIGN 17
4. STUDY POPULATION 19
4.1 Population (base) 19
4.2 Inclusion criteria 19
4.3 Exclusion criteria 19
4.4 Sample size calculation 20
5. TREATMENT OF SUBJECTS 21
5.1 Investigational product/treatment 21
5.2 Use of co-intervention 21
5.3 Escape medication 21
6. INVESTIGATIONAL PRODUCT 22
6.1 Name and description of investigational product(s) 22
6.2 Summary of findings from non-clinical studies 22
6.3 Summary of findings from clinical studies 22
6.4 Summary of known and potential risks and benefits 23
6.5 Description and justification of route of administration and dosage 23
6.6 Dosages, dosage modifications and method of administration 23
6.7 Preparation and labelling of Investigational Medicinal Product 23
6.8 Drug accountability 24
7. NON-INVESTIGATIONAL PRODUCT 25
8. METHODS 26
8.1 Study parameters/endpoints 26
8.1.1 Main study parameter/endpoint 26
8.1.2 Secondary study parameters/endpoints 26
8.1.3 Other study parameters (if applicable) 27
8.2 Randomisation, blinding and treatment allocation 28
8.3 Study procedures 28
8.3.1 Study flowchart 31
8.4 Withdrawal of individual subjects 31
8.4.1 Specific criteria for withdrawal (if applicable) 31
8.5 Replacement of individual subjects after withdrawal 32
8.6 Follow-up of subjects withdrawn from treatment 32
8.7 Premature termination of the study 32
9. SAFETY REPORTING 33
9.1 Temporary halt for reasons of subject safety 33
9.2 AEs, SAEs and SUSARs 33
9.2.1 Adverse events (AEs) 33
9.2.2 Serious adverse events (SAEs) 33
9.2.3 Suspected unexpected serious adverse reactions (SUSARs) 34
9.3 Annual safety report 34
9.4 Follow-up of adverse events 35
9.5 [Data Safety Monitoring Board (DSMB) / Safety Committee] 35
10. STATISTICAL ANALYSIS 36
10.1 Primary study parameter(s) 36
10.2 Secondary study parameter(s) 36
10.3 Other study parameters 37
10.4 Interim analysis (if applicable) 37
11. ETHICAL CONSIDERATIONS 38
11.1 Regulation statement 38
11.2 Recruitment and consent 38
11.3 Objection by minors or incapacitated subjects (if applicable) 38
11.4 Benefits and risks assessment, group relatedness 38
11.5 Compensation for injury 39
11.6 Incentives (if applicable) 39
12. ADMINISTRATIVE ASPECTS, MONITORING AND PUBLICATION 40

12.1 Handling and storage of data and documents 40
12.2 Monitoring and Quality Assurance 40
12.3 Amendments 40
12.4 Annual progress report 40
12.5 Temporary halt and (prematurely) end of study report 40
12.5 Public disclosure and publication policy 40
13. STRUCTURES RISK ANALYSIS 42
13.1 Potential issues of concern 42
13.2 Synthesis 42
14. REFERENCES 43

LIST OF ABBREVIATIONS AND RELEVANT DEFINITIONS

**ABC**	**Activity Based Costing**
**ABR**	**General Assessment and Registration form (ABR form), the application form that is required for submission to the accredited Ethics Committee; in Dutch: Algemeen Beoordelings- en Registratieformulier (ABR-formulier)**
**AE**	**Adverse Event**
**AR**	**Adverse Reaction**
**BIA**	**Budget Impact Analysis**
**CA**	**Competent Authority**
**CCMO**	**Central Committee on Research Involving Human Subjects; in Dutch: Centrale Commissie Mensgebonden Onderzoek**
**CDLT**	**Cisplatin Dose Limiting Toxicity**
**CRT**	**Chemoradiotherapy**
**CT**	**Computed Tomography**
**CTCAE**	**Common Terminology Criteria for Adverse Events**
**CV**	**Curriculum Vitae**
**C3**	**Third cervical vertebrae**
**DSMB**	**Data Safety Monitoring Board**
**ECOG**	**Eastern Cooperative Oncology Group**
**EU**	**European Union**
**EudraCT**	**European drug regulatory affairs Clinical Trials**
**FDG-PET**	**Fluorodeoxyglucose-Positron Emission Tomography**
**GCP**	**Good Clinical Practice**
**GDPR**	**General Data Protection Regulation; in Dutch: Algemene Verordening Gegevensbescherming (AVG)**
**HI**	**Hounsfield Unit**
**HNSCC**	**Head and Neck Squamous Cell Carcinoma**
**IB**	**Investigator's Brochure**
**IC**	**Informed Consent**
**IMP**	**Investigational Medicinal Product**
**IMPD**	**Investigational Medicinal Product Dossier**
**LA-HNSCC**	**Locally Advanced Head and Neck Squamous Cell Carcinoma**
**L3**	**Third lumbar vertebra**
**METC**	**Medical research ethics committee (MREC); in Dutch: medisch-ethische toetsingscommissie (METC)**
**MRI**	**Magnetic Resonance Imaging**

**NCI**	**National Cancer Institute**
**NWHHT**	**Dutch Head and Neck Society**
**NZA**	**Dutch Healthcare Authority**
**OS**	**Overall Survival**
**PCQ**	**Productivity Cost Questionnaire**
**Sponsor**	**The sponsor is the party that commissions the organisation or performance of the research, for example a pharmaceutical company, academic hospital, scientific organisation or investigator. A party that provides funding for a study but does not commission it is not regarded as the sponsor, but referred to as a subsidising party.**
**SUSAR**	**Suspected Unexpected Serious Adverse Reaction**
**TNM**	**Tumor Node Metastasis**
**UAVG**	**Dutch Act on Implementation of the General Data Protection Regulation; in Dutch: Uitvoeringswet AVG**
**WMO**	**Medical Research Involving Human Subjects Act; in Dutch: Wet Medisch-wetenschappelijk Onderzoek met Mensen**

SUMMARY

Rationale: In the Netherlands, 3160 patients were diagnosed with HNSCC in 2018. Two-thirds of HNSCC patients present with locally advanced disease (LA-HNSCC). The standard of care consists of intravenous cisplatin concurrently given with conventional external beam radiotherapy (chemoradiotherapy, CRT). High cumulative cisplatin dose is associated with better outcome. The most commonly used scheme is three-weekly high dose cisplatin of 100mg/m^2. Though effective in terms of overall survival (OS) and loco-regional control (LRC), high rates of severe acute events lead to early cessation of therapy in up to 40% of patients and cause decrease in local control and survival. Furthermore in 13% of the patients late toxicity is reported, which leads to permanent comorbidity. Currently, patients at risk for this toxicity cannot be accurately identified upfront. Another commonly used scheme is weekly low dose cisplatin of 40 mg/m^2 concurrently given with radiotherapy (RT). In practice, this scheme is also widely accepted as standard of care as it results in less acute toxicity even though the high level of evidence using meta-analysis prefers the high-dose scheme on a group level. Currently, it is assumed that, in a not yet identified specific subset of patients at risk for toxicity following the high-dose three-weekly scheme, weekly concurrent cisplatin might be more appropriate, leading to better tolerance, less toxicities and to a higher cumulative cisplatin dose. It has been shown previously that patients with low skeletal muscle mass (SMM) were more than three times more likely to develop cisplatin dose limiting toxicity (CDLT) compared to patients with normal SMM (44.3% vs. 13.7%); consequently, compliance (no CDLT) rate to planned chemotherapy scheme was 55.7% and 86.3%. Moreover, patients with CDLT also had a lower OS. This leads to the hypothesis that particularly patients with low SMM may benefit from weekly low dose cisplatin concurrent RT, leading to better compliance compared to the high dose schedule. We hypothesize that in LA-HNSCC patients with low SMM, receiving weekly low dose cisplatin concurrent RT can improve compliance rate to planned chemotherapy scheme from 55.7% to 86.3%.

Objective: To investigate if the use of weekly low dose cisplatin increases compliance to the planned chemotherapy scheme in LA-HNSCC patients with low SMM to a level of compliance to three-weekly high dose cisplatin of patients without low SMM.

Study design: In a multicenter prospective randomised low intervention clinical trial the compliance of weekly low dose compared to three-weekly high dose cisplatin with concurrent RT in seventy LA-HNSCC patients with low SMM will be investigated. To assure the inclusion of seventy low SMM patients, a total of 129 LA-HNSCC patients should be included according to the incidence rate of low SMM in this population.

Study population: Patients who are planned for concomitant CRT with cisplatin as standard of care treatment will be asked to participate in this study. SMM is measured on routinely performed CT or MRI scans pretreatment and seventy patients with low SMM will be randomised between weekly low dose and three-weekly high dose cisplatin.

Intervention: Randomisation between high- (100 mg/^{m2} three-weekly) and low dose (40 mg/m^2 weekly) treatment. Cisplatin treatment itself and concomitant radiotherapy are not part of the intervention, but part of standard treatment.

Main study parameters: The primary outcome of this randomised low intervention clinical trial is compliance to the planned chemotherapy scheme in patients with low SMM. Secondary outcomes of this study are toxicity, cumulative cisplatin dose, time to recurrence, overall survival, costs, quality of life and patient's preference. Toxicity e.g., adverse events (AE) will be assessed using the Common Terminology Criteria for Adverse Events (CTCAE) v5.0 formulated by the National Cancer Institute (NCI).

Nature and extent of the burden and risks associated with participation, benefit and group relatedness: Burden to patients is limited to completion of four questionnaires for five times, which will take about 30 minutes, and therefore it is likely that most patients will agree to participate. There will be no need for extra diagnostic procedures, because CT at cervical level is standard pre-treatment procedure in LA-HNSCC. Moreover each patient receives RT daily, so no additional hospital visit is necessary: Radiotherapy will be given daily during 7 weeks. Chemotherapy will be given three or seven times, depending on the randomisation group, on a day radiotherapy is planned as well. For low SMM patients, this study may serve as a basis for increase in compliance to therapy and might increase survival and LRC, since early discontinuation of CRT increases risks at recurrence of disease.

1. INTRODUCTION AND RATIONALE

Worldwide an estimated 900.000 new cases of head and neck squamous cell carcinoma (HNSCC) developed in 2018. This is approximately 5% of all new cancer cases.[1] In the Netherlands, 3160 patients are diagnosed with HNSCC in 2018. Two-thirds of HNSCC patients present with advanced disease at diagnosis (large tumor, regional metastasis and/or distant metastasis). Concomitant cisplatin based chemoradiotherapy (CRT) is the preferred treatment for patients with unresectable locally advanced head and neck squamous cell carcinoma (LA-HNSCC) and functional inoperable LA-HNSCC aiming organ and function preservation. The most common cisplatin dosage is 100mg/m^2 every 3 weeks for 3 cycles combined with 7 weeks of conventional radiotherapy, consisting of 70Gy in 35 fractions.[2–8] An alternative frequently used scheme is weekly low-dose (seven cycles of 40mg/m^2) cisplatin with concurrent radiotherapy. Cisplatin is routinely used in the curative treatment to enhance the antitumor activity of irradiation.

Among various proposed treatment schedules, differing in frequency, dose, and route of administration, there is level 1 evidence for significant improvement in loco-regional control (LRC) and overall survival achieved by three-weekly high dose (three cycles of 100mg/m^2) intravenous cisplatin given concurrently with conventional external beam radiotherapy (RT) compared to RT alone.[9,10] Despite indisputable efficacy, high rates of severe acute and late adverse events remain of concern. Most common acute adverse events (AE) comprise of mucositis, dermatitis, emesis, anemia, leukopenia, thrombocytopenia and acute kidney injury.[11,12] Late AE, compromising quality of life, consist of dysphagia, xerostomia, ototoxicity, radionecrosis and hypothyroidism.[13] In fact, even though extensive pre-treatment assessment and selection is performed, due to unacceptable systemic and local toxicities, up to 40% of patients fail to comply to all three planned cycles of high-dose cisplatin, which decreases local control and overall survival (3-year overall survival from 72% to 52%) in individual patients.[14] Besides, severe late toxicity has been reported in 13% of patients and can lead to permanent comorbidity. [14] A systematic review pointed out a significant association between cumulative cisplatin dose and overall survival (OS).[15] Also Spreafico et al. found in a pooled analysis of 659 patients with LA-HNSCC a survival benefit with cisplatin above 200mg/m^2.[16] This emphasizes the necessity of a feasible scheme for all patients, to assure that the patient will be able to finish the whole planned treatment. Although Jacinto et al. concluded in a recent systematic review that three-weekly cisplatin schedule should remain the standard of care for LA-HNSCC, they suggest a specific subset of patients (not yet identified) in whom weekly concurrent cisplatin is more appropriate and a higher cumulative cisplatin dose can be achieved, which is in line with recommendations of other recently published studies.[9,14,17–21] In a recent systematic review Szturz et al. compared the standard, three-weekly high-dose (three cycles of 100mg/m^2) cisplatin with concurrent RT and its alternative, weekly low-dose (seven cycles of 40mg/m^2) cisplatin with concurrent RT in LA-HNSCC. They found that treatment adherence was higher in the weekly schedule (88%) compared to the three-weekly schedule (71%; p=0.0017), however the exact sample size of each study and specific survival rates are unclear and no statistical difference in survival was observed for the total population.[14]

To our knowledge no adequate randomised controlled trials have been performed in low SMM patients with primary chemoradiation receiving three-weekly 100 mg/m^2 or weekly 40 mg/m^2 cisplatin. Several randomised controlled trials showed superiority of high-dose three-weekly cisplatin over a lower weekly dose of cisplatin in patients with adjuvant chemoradiation. 1). Noronha et al. showed superiority of three-weekly high-dose cisplatin over weekly low-dose (30 mg/m^2) in the cumulative 2-year LRC (73.1% versus 58.5%) but a higher toxicity rate in the high-dose arm (84.6% versus 71.6%). No significant difference in compliance was found (p = 0.1). In this study relatively fit LA-HNSCC patients were included, which might cause underestimation of incompliance to therapy in low SMM patients thus leading to a wrong conclusion in case of patients with low SMM. Also, the weekly treatment was underdosed (30 instead of 40 mg/m2).[22] 2). Tsan et al. showed a higher compliance in the post-operative 100 mg/m^2 three-weekly cisplatin group compared to 40 mg/m^2 weekly cisplatin group. However, they do not report anything on skeletal muscle mass, performance status or body mass index. Thus, there might be a significant difference between baseline characteristics of the intervention and control group, contributing to the positive results in the three-weekly cisplatin group. [23] 3). Quon et al. showed that addition of very low-dose (20 mg/m^2) to RT did not improve survival.[24] Despite lack of level I evidence, due to high toxicity rate, weekly low-dose cisplatin (40 or 30 mg/m^2) has substituted three-weekly cisplatin CRT in common practice and trials.[11,25–32] Some institutes in the Netherlands, already implemented weekly cisplatin as standard CRT for susceptible groups. This is in line with the recent National Comprehensive Cancer Network guidelines, which mentions that dosing schedules of cisplatin other than three-weekly 100 mg/m^2 are also efficacious.[21] In the European Society for Medical Oncology (ESMO) guidelines for head and neck cancers, the use of three-weekly 100 mg/m^2 cisplatin is advised for fit patients (level I evidence), but weekly 40 mg/m^2 cisplatin is also a proper treatment as level II evidence is available. [7] In the Dutch Richtlijnendatabase weekly 40 mg/m^2 cisplatin is mentioned as an alternative of three-weekly 100 mg/m^2 cisplatin.[33] Moreover a weekly dose of 40 mg/m^2 is sometimes used as standard treatment in several hospitals in the Netherlands. We would like to emphasize that both schemes can be considered as clinical equipoise according to expert opinion. In both schemes we aim to give a cumulative dose of more than 200 mg/m^2 and both schemes are used in daily clinical practice.

A retrospective study in patients with LA-HNSCC undergoing CRT with three-weekly cisplatin found an association between low skeletal muscle mass (SMM) and the occurrence of cisplatin dose limiting toxicity (CDLT). [34] Total (whole body) SMM can reliably be estimated based on measurement of the cross-sectional area of the sternocleidomastoid muscle and paravertebral muscles on the level of the third cervical vertebrae (C3) on routinely performed CT or MRI. This method has been validated and appeared to be robust when comparing it to the common method measuring the muscle area at the level of the third lumbar vertebrae (L3).[35,36] CDLT was defined as cisplatin induced toxicity causing either $\geq$4 days postponement of the treatment, $\geq$50% dose-reduction or termination of therapy before the third cycle. [34] Patients with low SMM were more than three times more likely to develop CDLT compared to patients with normal SMM (44.3% vs. 13.7%); consequently, compliance (no CDLT) rate to planned chemotherapy scheme was 55.7% and 86.3%. Moreover, patients experiencing CDLT had

a lower overall survival than patients who did not (mean 36.6 vs. 54.2 months, p = 0.038). [34] Cisplatin possibly distributes mainly to the fat-free mass, of which SMM is the largest component, and thereby a higher and more toxic peak dosage might be reached in patients with low SMM receiving high dose cisplatin. [34] Since early discontinuation of therapy automatically leads to a reduced cumulative cisplatin dose, which is associated with lower overall survival, it can be anticipated that particularly patients with low SMM might benefit from weekly low dose cisplatin based concurrent CRT to achieve an adequate cumulative dose comparable to patients with normal SMM. In this multicenter prospective randomised low intervention clinical trial, we aim to investigate if weekly low dose cisplatin can increase the compliance to the planned chemotherapy scheme in patients with low SMM to a level of compliance to three-weekly high dose cisplatin of patients without low SMM.

2. OBJECTIVES

In this multicenter prospective randomised low intervention clinical trial, we aim to investigate if the use of weekly low dose cisplatin can increase the compliance to the planned chemotherapy scheme in LA-HNSCC patients with low SMM to a level of compliance to three-weekly high dose cisplatin of patients without low SMM. We hypothesize that in LA-HNSCC patients with low SMM undergoing concurrent primary CRT weekly with low dose cisplatin compliance rate to planned chemotherapy scheme improves from 55.7% to 86.3%. The primary outcome of this study is the difference in compliance (defined as absence of CDLT) rate to the proposed cisplatin scheme between weekly low dose cisplatin and three-weekly high dose cisplatin in patients with low SMM. Secondary outcomes of this study are treatment cumulative cisplatin dose, acute and late adverse events (AE's), time to recurrence, 2-years survival, quality of life, costs and patient's preference.

Primary Objective:

- To compare the compliance rate to proposed cisplatin scheme between weekly low dose cisplatin or three-weekly high dose cisplatin in patients with low SMM.

Secondary Objective(s):

- To investigate whether weekly low dose cisplatin can increase the compliance to the planned chemotherapy scheme in LA-HNSCC patients with low SMM to a level of compliance to three-weekly high dose cisplatin of patients without low SMM.
- To investigate treatment cumulative cisplatin dose in patients with low SMM receiving weekly low dose or three-weekly high dose cisplatin.
- To investigate acute and late adverse events/toxicity in patients with low SMM receiving weekly low dose or three-weekly high dose cisplatin.
- To investigate time to recurrence in patients with low SMM receiving weekly low dose or three-weekly high dose cisplatin.
- To investigate 2-years survival in patients with low SMM receiving weekly low dose or three-weekly high dose cisplatin.
- To investigate quality of life in patients with low SMM receiving weekly low dose or three-weekly high dose cisplatin.
- To investigate costs in care for patients with low SMM receiving weekly low dose or three-weekly high dose cisplatin.
- To investigate patient's preference of treatment in patients with low SMM receiving weekly low dose or three-weekly high dose cisplatin.

3. STUDY DESIGN

In this multicenter prospective randomised low intervention clinical trial, the compliance of weekly low dose cisplatin with concurrent radiotherapy in LA-HNSCC patients with low SMM will be investigated. SMM will be measured on routinely performed (PET-)CT or MRI scans before the multidisciplinary head and neck cancer meeting by the treating physician. Patients who are found eligible by the multidisciplinary team and are planned for cisplatin based concurrent CRT will be asked to participate in this study by the investigator. Seventy patients with low SMM will be randomised between weekly low dose and three-weekly high dose cisplatin. To assure the inclusion of seventy patients with low SMM, 129 patients in total are needed as explained in paragraph 4.4. Therefore 59 patients with normal skeletal muscle mass will be asked to participate as well. They will receive the most standard cisplatin scheme for the centre they are admitted to specifically and will be included in follow-up. Baseline characteristics and outcomes will be collected for all patients.

SMM will be estimated using a validated technique based on measurement of the cross-sectional area of the sternocleidomastoid muscle and paravertebral muscles on the level of C3 on routinely performed CT or MRI. In patients who underwent fluorodeoxyglucose-positron emission tomography(FDG-PET)/CT as part of the diagnostic work-up, as a control of SMM measurement at the level of C3, SMM will also be measured at the level of L3, which is the most commonly used method in medical literature as a control of SMM measurement at the level of C3.

Based on the cut-off value calculated by Wendrich et al., low SMM is defined as skeletal muscle mass $\leq$43,3 cm2/m^2.[34] Patients with low SMM will be randomised between weekly low dose cisplatin and three-weekly high dose cisplatin with concurrent RT. The rate of compliance to proposed cisplatin scheme will be compared between three groups: patients with low SMM with weekly low dose cisplatin, patients with low SMM and three-weekly high dose cisplatin and the rest group of normal SMM receiving the local standard scheme, which is three-weekly 100 mg/m^2 cisplatin in the UMC Utrecht, Noordwest Ziekenhuisgroep, Leiden University Medical Center, and VUmc, and weekly 40 mg/m^2 cisplatin in the NKI-AVL.

During chemoradiation, all patients will be frequently seen by their radiotherapist and oncologist. It is conform standard procedures to document toxicities and the researcher will collect data and add it to the database. Follow-up will consist of clinical examination every two months in the first year and every three months in the second year, for at least 24 months after treatment which is conform standard procedures. Low SMM patients will be asked to answer the EORTC QLQ-C30, EORTC-QLQ-H&N35, EQ-5D-5L and PCQ questionnaires before and 3, 6, 12 and 24 months after end of CRT. Normal SMM patients will be asked to answer the EORTC QLQ-C30, EORTC-QLQ-H&N35, EQ-5D-5L questionnaires before and 3, 6, 12 and 24 months after CRT. Questionnaires will initially be send by e-mail using Castor (EDC), but on patient's or treating physicians request paper questionnaires are available as well. Paper questionnaires will be transmitted to Castor EDC by the

executive researcher or (delegated by) the principal investigator. Patients' experience will be explored three months after CRT by qualitative research using semi-structured interviews. Semi-structured interviews will be analyzed by two investigators using thematic descriptive analyses. A cost-effectiveness analysis will be performed to compare three-weekly and weekly cisplatin treatment. Time interval between inclusion and end of treatment patient will be approximately two months, total time of follow-up will be 24 months after end of treatment. After follow-up, 6 months will be given for analysis and final report. The end of treatment is defined as the day of the last radiation session and is consistent for all groups.

4. STUDY POPULATION

4.1 Population (base)

Patients with LA-HNSCC who are planned for cisplatin based primary concurrent CRT (as standard of care) in one of the participating centers of the Dutch Head and Neck Society will be asked to participate in this study. In the Netherlands yearly 250 LA-HNSCC are treated with primary CRT and in a previous study 54.5% patients had low SMM.[34]

SMM will be estimated based on measurement of the cross-sectional area of the sternocleidomastoid muscle and paravertebral muscles on the level of the third cervical vertebrae (C3) on routinely performed CT or MRI. In patients who undergo FDG-PET/CT as part of the routine diagnostic work-up, as a control of SMM measurement at the level of C3, SMM will also be measured at the level of the third lumbar vertebra (L3), which is the most commonly used method in medical literature as a control of SMM measurement at the level of C3.

Seventy patients with low SMM will be randomised between low-dose weekly and high-dose three-weekly cisplatin therapeutic scheme with concurrent radiation. 59 patients with normal SMM will also be asked to participate in this study and will receive the standard of care of the centre they are admitted to (either three-weekly 100 mg/m^2 or weekly 40 mg/m^2 cisplatin). If 129 patients are included, but the minimum of 70 patients with low SMM is not yet reached, we will include additional patients until 70 patients with low SMM are included, in order to create sufficient power for statistical analysis. See paragraph 4.4 for the exact calculation.

4.2 Inclusion criteria

In order to be eligible to participate in this study, a subject must meet all of the following criteria:

- considered, eligible and planned for primary cisplatin CRT by treating physician;
- eighteen years of age or older;
- sufficient understanding of Dutch and medical consequences to give informed consent.

4.3 Exclusion criteria

A potential subject who meets any of the following criteria will be excluded from participation in this study:

- mentally disabled or patients with significantly altered mental status that would prohibit understanding and giving informed consent;
- a history of bilateral lymph node dissection in the neck and no available (PET-)CT scan of the third lumbar vertebra;
- an absolute contraindication for cisplatin as defined by the treating physician, including relevant pre-existing kidney insufficiency, clinically apparent vascular disease (for example

claudicatio intermittens), clinically relevant perceptive deafness, serious neuropathy and poor performance score;

- an absolute contraindication for high dose three-weekly cisplatin 100 mg/m^2 as defined by the treating physician;
- interval between diagnostic scan and planned CRT >2 months;
- cisplatin CRT planned as non-primary or induction treatment.

4.4 Sample size calculation

In the retrospective study by Wendrich et al., out of 112 patients 30.4% experienced CDLT (meaning compliance to chemotherapy regimen was 69.6%). Using a cut-off 43.2 cm^2/m^2, 54.5% patients had low SMM. Patients with low SMM experienced CDLT more frequently than patients with normal SMM (44.3% vs. 13.7%, $p < 0.001$).[34] Thus compliance rates to chemotherapy regimen were 55.7% and 86.3%, respectively. We hypothesize therefore that in patients with low skeletal muscle mass and weekly low dose cisplatin, also 86.3% compliance will be reached. If we compare this compliance to an expected compliance of 55.7% in the arm of with a three-weekly high dose cisplatin, 33 patients with low SMM per arm are needed to show that the compliance in the weekly low dose cisplatin scheme is statistically significantly better than the threeweekly high dose scheme (two-sided alpha 0.05 and power 80%). With an expected drop-out of 5% 70 patients with low SMM are needed. Power calculations were done using PASS software. Since 54.5% of patients undergoing cisplatin based concurrent CRT have low SMM, to find the 70 patients with low SMM a total of 129 LA-HNSCC patients undergoing cisplatin based concurrent CRT are needed.

5. TREATMENT OF SUBJECTS

5.1 Investigational product/treatment

In this multicenter prospective randomised low intervention clinical trial patients with low SMM are randomised for three-weekly high dose (three cycles of 100mg/m^2) or weekly low dose (seven cycles of 40mg/m^2) cisplatin intravenously given with concurrent radiotherapy (35x 2 Gy; five times weekly with a total of 7 weeks). We consider both schems as clinical equipoise, according to expert opinion.[16,21] As previously mentioned, we aim to give a cumulative dose of more than 200 mg/m^2 and both schemes are used in daily clinical practice. All patients will be provided with supportive care according to local standard. Adequate pre- and posthydration, using sodium-chloride 0.9% solution with electrolyte suppletion will be performed. Furthermore, patients will receive anti-emetics, laxatives, diuretics and supportive care for treatment of side effects according to local standard. No experimental medications will be permitted while the patient is participating in this study.

5.2 Use of co-intervention

Co-interventions that might be used will be conform standard cisplatin based concurrent CRT protocol so therefore there will be no differences between the groups.

5.3 Escape medication

NA

6. INVESTIGATIONAL PRODUCT

For more detailed information regarding the investigational product (Cisplatin), we would like to refer to the attached SPC.

6.1 Name and description of investigational product(s)

Cisplatin in locally-advanced head and neck cancer consist of 100mg/m^2 every three weeks during three cycles, but the National Comprehensive Cancer Network guidelines mentions that dosing schedules of cisplatin other than three-weekly 100 mg/m^2 are also efficacious. [21] According to the European Society of Medical Oncology guidelines, cisplatin is the preferred treatment for all patients with LA-HNSCC who are not fit enough for surgery of in whom functionality of the larynx should be preserved. Furthermore there is level II evidence for the efficacy of weekly 40 mg/m^2 cisplatin.[7]

The intervention arm will receive 40 mg/m2 cisplatin weekly during seven weeks. The control arm will receive 100 mg/m2 cisplatin every three weeks for three cycles over a period of seven weeks. Administration is standardized for cisplatin and consists of intravenous cisplatin solution slowly (four hours) injected.

The combination of cisplatin with aluminium should be avoided, as stated by the Summary of Product Characteristics (See: Paragraph 6.2 on page 13). Preparation and labelling should be done conform hospital guidelines regarding chemotherapeutics. The standard procedures for chemotherapeutics should be followed. There is no need for the patient to return the medicine, because administration will take place in the hospital.

6.2 Summary of findings from non-clinical studies

Not applicable since cisplatin has been widely used in clinical practice over decades. It is registered in the Netherlands as a standard of care treatment (RVG 104068).

6.3 Summary of findings from clinical studies

Cisplatin is the preferred treatment for patients with unresectable LA-HNSCC or a functional larynx. The most common dosage is cisplatin 100mg/m^2 combined with 7 weeks of conventional radiotherapy, consisting of 70Gy in 35 fractions.[2–5] Unfortunately high rates of severe acute and late adverse events remain of concern. Most common acute adverse events (AE) comprise of mucositis, dermatitis, emesis, anemia, leukopenia, thrombocytopenia and acute kidney injury.[11,12] Late AE, compromising quality of life, consist of dysphagia, xerostomia, ototoxicity, radionecrosis and hypothyroidism.[13]

To our knowledge three recent large randomized controlled trials compared cisplatin weekly low-dose to three-weekly high dose schemes. 1). Noronha et al. showed superiority of three-weekly high-dose cisplatin over weekly low-dose (30 mg/m^2) in the cumulative 2-year LRC (73.1% versus 58.5%) but a higher toxicity rate in the high-dose arm (84.6% versus 71.6%). No significant difference in compliance was found ($p = 0.1$). In this study relatively fit LA-HNSCC patients were included, which might cause

underestimation of incompliance to therapy in low SMM patients thus leading to a wrong conclusion in case of patients with low SMM. Also, the weekly treatment was underdosed (30 instead of 40 mg/m2).[22] 2). Tsan et al. showed a higher compliance in the post-operative 100 mg/m^2 three-weekly cisplatin group compared to 40 mg/m^2 weekly cisplatin group. However, they do not report anything on skeletal muscle mass, performance status or body mass index. Thus, there might be a significant difference between baseline characteristics of the intervention and control group, contributing to the positive results in the three-weekly cisplatin group. [23] 3). Quon et al. showed that addition of very low-dose (20 mg/m^2) to RT did not improve survival.[24] Despite lack of level I evidence, due to high toxicity rate, weekly low-dose cisplatin (40 or 30 mg/m^2) has substituted three-weekly cisplatin CRT in common practice and trials.[11,25–32] This is in line with the recent National Comprehensive Cancer Network guidelines, which mentions that dosing schedules of cisplatin, other than three-weekly 100 mg/m^2 are efficacious. [21] In an ongoing phase II/III study (JCOG1008), preliminary available results suggest that weekly 40mg/m^2 cisplatin post-operative is an appropriate and safe dose of cisplatin in high-risk patients improving OS, (local) relapse free survival and decreasing acute toxicities.[37]

Thus weekly cisplatinum (40mg/m^2) with concurrent RT, is commonly used in practice but not investigated in a large randomized controlled trial in patients with low skeletal muscle mass. As a level 2B category evidence, it is used anyway in practice.[7]

6.4 Summary of known and potential risks and benefits

See explanatory text of chapter 6.1, 6.3 and the enclosed SPC.

6.5 Description and justification of route of administration and dosage

See explanatory text of chapter 6.1, 6.3 and the enclosed SPC.

6.6 Dosages, dosage modifications and method of administration

The intervention arm will receive 40 mg/m^2 cisplatin weekly during seven weeks. The control arm will receive 100 mg/m^2 cisplatin every three weeks for three cycles in the UMCU, Noordwest Ziekenhuisgroep, Leiden University Medical Center, and VUmc. Patient with a normal SMM in the AVL, will receive 40 mg/m^2 cisplatin weekly during seven weeks, since this is the standard treatment for the AVL. Administration is standardized for cisplatin and consists of intravenous cisplatin solution slowly (approximately four hours) injected.

6.7 Preparation and labelling of Investigational Medicinal Product

The investigational product has a marketing authorisation and is used in the authorised form for the authorised indication. Therefore, the investigational products are used from commercial stock and preparation and labelling are performed within the pharmacy department of the participating hospitals. No specific labelling is performed in this study. Administration will take place according to local guidelines and in line with recommendations in the National Comprehensive Cancer Network and European Society of Medical Oncology guidelines.[7,21]

6.8 Drug accountability

Together with the local pharmacy, drug accountability according to the GCP guidance will be performed. The pharmacy will not label the IMP as explained in 6.7. Preparation for administration of cisplatin will be performed according to local standard pharmacy procedures. For each administration, patient-named drug accountability, as always done for chemotherapeutic agents, will be done, also according to local standard pharmacy procedures.

7. NON-INVESTIGATIONAL PRODUCT

NA

8. METHODS

8.1 Study parameters/endpoints

8.1.1 Main study parameter/endpoint

The primary outcome parameter is compliance (non CDLT) rate to the proposed cisplatin scheme. Compliance to chemotherapy is defined as the absence of CDLT. CDLT is defined as any toxicity resulting in a cisplatin dose-reduction of ≥50%, a postponement of treatment of ≥4 days or a definite termination of cisplatin after the first or second cycle of therapy. For weekly cisplatin regimens CDLT is defined as skipped cisplatin treatments or a definite termination of cisplatin before all scheduled cycles of therapy are given.

8.1.2 Secondary study parameters/endpoints

Secondary outcome parameters are adverse events/toxicity, cumulative cisplatin dose, time to recurrence, 2-year overall survival, costs, quality of life and patient's preference. The main oncological outcome parameters are time to recurrence and survival. Clinically relevant treatment related toxicity parameters, including specific toxicity that results in significant (grade 3 or 4) toxicity, treatment de-escalation or termination, will be recorded by the treating medical oncologist. Toxicity will be scored according to the Common Terminology Criteria for Adverse Events (CTCAE) guidelines, v5.0. [12]

Quality of life questionnaires

The following questionnaires will be used to measure quality of life: EORTC QLQ-C30, EORTC-QLQH& N35 and EQ-5D-5L. Global quality of life is assessed with the EORTC-QLQ-C30 global measure. The EORTC-QLQ-H&N35 is designed to be head and neck cancer-specific, multidimensional in structure, appropriate for self-administration and applicable across a range of cultural settings. The EQ-5D-5L is a standardized instrument can be used as a quantitative measure of health outcome that can be used in a wide range of health conditions and treatments, and reflects the patient's own judgement. Questionnaires are asked to fill out before and 3, 6, 12 and 24 months after CRT. Standard scoring methods are applied to quality-of-life questionnaires. All scores are normalized, ranging from 0 to 100, and transformed to unweighted summated scales in which higher scores indicates better health. Separate comparisons are made at each time point. Unadjusted p values are used. On the EORTC questionnaires a 10-point difference in scores was considered to be clinically relevant.

Cost analysis

Substitution of weekly low dose cisplatin for three-weekly high dose cisplatin in patients treated with concurrent CRT is expected to result in cost-savings and an increase in quality of life due to reducing the number of complications. A detailed analyses of cost differences for low SMM patients with weekly low dose cisplatin and standard of care (three-weekly high dose cisplatin) demands a detailed collection of all health care consumed by these patients in relation to the intervention and complications. In the prospective cohort we will collect all

health care consumed by patients in each of the three different groups with a follow-up of 12 months. All data will be collected from the electronic patient files in each different hospital and will be collected using respective units for each sort of health care consumption (hospital stay per day, cost per dose, etc.). As indicated, quality of life will be measured before and 3, 6, 12 and 24 months after CRT using the EQ-5D-5L. Productivity loss of low SMM patients will be collected during the study at baseline and at 12 months after CRT using the productivity cost questionnaire (PCQ). This will only be done for the low SMM group, since our main interest is the difference between the two chemotherapy schemes in the low SMM group. All different units of care consumed will be linked to their respective unit costs. Reimbursement prices issued by the Dutch Healthcare Authority (NZA) and national reference prices will be used for this assessment as outlined in current Dutch pharmaco-economic guidance.

In addition to total health care consumption of these patients it is essential to perform micro-costing of CRT treatment to have detailed insight in the additional costs. We therefore aim to perform a micro-costing study using the activity based costing (ABC) method. To link both costs and effects we aim to develop a decision-tree completely outlining all probabilities of having complications (CDLT resulting in non-compliance) and their respective costs and effects (quality of life) in each of the three different groups. Outcome measure will be incremental costs per quality adjusted life year. Moreover, complete sensitivity analysis (both deterministic as well as probabilistic) will be carried out to have detailed insights into the impact of uncertainty on our outcome measures. In addition to a cost-effectiveness analyses we aim to perform a budget impact analysis (BIA) as well. The BIA adheres to the Zorginstituut guidelines and applies the perspectives: societal, health insurance/third party payer and health care (Budgetair Kader Zorg (BKZ)). The BIA Prices will be linked to perspectives: societal-CEA based prices, BKZ-average rates according to NZa, for health insurance perspective also NZa average rates and, for example, for a local health care provider perspective specific passenger rates ('passanten tarieven'). The BIA will be assessed through (decision analytical) modelling and analyzed in a probabilistic way.

8.1.3 Other study parameters (if applicable)

To allow for comparison with the recent nation-wide Dutch Head and Neck Society audit, the same characteristics and potential predictive factors will be scored.

Collected patient characteristics are gender, age, weight, stature (length), smoking history, use of alcohol, loss of weight, Eastern Cooperative Oncology Group (ECOG performance status), medical history regarding heart, lung, diabetes mellitus, oncology and nephrology, grip strength (if available), comorbidity (ACE-27 and Charlson Comorbidity Index), Tumor Node Metastasis (TNM) classification, tumor localisation, estimated Glomerular Filtration Rate (eGFR), serum creatinine, neutrophil count, platelet count, leukocyte count, lymphocyte count, monocyt count and hemaglobin level, baseline audiometry results and treatment plan (including use of co-medication).[38]

8.2 Randomisation, blinding and treatment allocation

When the patient has signed informed consent, patients with low SMM will berandomised by the investigator for three-weekly high dose (three cycles 100mg/m^2) or weekly low dose cisplatin (seven cycles 40mg/m^2). Allocation is performed by a central office on-site computer combined with allocations kept in a locked, unreadable computer file that investigators can assess only after the characteristics of an enrolled patients are entered. A stratified permuted-block procedure will randomise patients to the groups on a 1:1 ratio. Strata comprises center and two groups according to AJCC staging:

1) Stage I-III
2) Stage IV.

Neither patients, investigators nor office personnel are blinded to the group chosen by the allocation procedure.

8.3 Study procedures

All patients diagnosed with an indication for primary CRT, will be identified and screened for eligibility at a weekly multidisciplinary head and neck oncology meeting. SMM will be measured on routinely performed pre-treatment (PET-)CT or MRI of the head and neck. During the multidisciplinary meeting, it will be decided whether the patient has an indication for primary CRT. If the patient is considered for cisplatin CRT, the investigator will inform the patient about the study and asked to sign informed consent. Patients with normal or high SMM (>43.2 cm^2/m^2) will receive the standard cisplatin scheme, which differs per centre. This will either be three-weekly high-dose cisplatin of 100 mg/m^2 or a weekly low-dose cisplatin of 40 mg/m^2. Patients with a low SMM $\leq$43.2 cm^2/m^2 will be randomised for either low- or high-dose cisplatin, respectively 40 mg/m^2 or 100 mg/m^2. Dependent on each centre, the local pharmacy or the local pharmacy and study pharmacy will be involved since treatment with cisplatin is standard of care and the planned cumulative dosage for both groups will be comparable.

The 100 and 40 mg/m^2 schemes differ in dose and frequency, but also in administration: three times hospital admission versus 7 day visits at the (outpatient) clinic. Because every patient receives radiotherapy daily (which is part of the standard of care), no additional hospital visits are required. In case of CDLT, the treating physician will decide which therapy is most suitable to the patient. It is conform standard guidelines to switch to different type of therapy when cisplatin has become too toxic. There will be no cross-over from the high to low-dose group, since this is against standard of care in which cisplatin therapy is stopped and carboplatin is given instead. The data will still be eligible for analysis in this research. During treatment, the patient will frequently be seen by the treating radiotherapist and oncologist. Adverse events, such as toxicities and CDLT are reported in the EPR according to standard hospital guidelines. Two to three days after each cisplatin dose, the kidney function will be determined. The investigator will collect clinical relevant data for each week of treatment. Data collected include CDLT, cause and type of acute toxicity, grade of toxicity according to Common

Terminology Criteria for Adverse Events (CTCAE) v5.0 guidelines (CTCAE), treatment to reduce toxicity and/or side effects and change of treatment strategy (e.g. switch from cisplatin to carboplatin). During follow-up, the patient is seen by the treating physician according to a standardized scheme. The investigator will collect data at 3, 6, 12 and 24 months after end of CRT, with a possible delay of maximum 6 weeks. Data consist of early and late toxicities, recurrence, residual disease, lost-to-follow-up and death.

Relevant and related early and late toxicities during and after CRT will be classified according to CTCAE v5.0 criteria with additional datacollection for toxicities concerning renal function, bone marrow dysfunctioning, hearing loss and neurotoxicity. Other adverse events not specified by CTCAE v5.0 will be described by the investigator in terms of symptoms, severeness, treatment and outcome. For each toxicity/event, date of start symptoms, date of diagnosis, treatment type, date of start and date of end of treatment will be collected. It will be stated whether the symptoms resolved, remained or improved and when this happened.

Nephrotoxicity: In case nephrotoxicity occurs, the eGFR, creatinine, and if available magnesium before CRT, during kidney dysfunction and at last moment of follow-up will be collected and compared as well.

Ototoxicity: When the treating physician suspects hearing loss in a patient, additional audiologic exam three months after CRT is recommended according to standard practice. Audiometry results and symptoms such as otitis, hearing loss and tinnitus, will be collected in the database to assess possible ototoxicity. Grading will be done using CTCAE v5.0 and the local preferred grading system.[12,39]

Neurotoxicity: Neurologic symptoms will be assessed as either peripheral motor or sensory neuropathy. Other neurologic symptoms, possible related to HNSCC treatment, not classified as peripheral neuropathy will be described.

Bone marrow dysfunctioning: To assess degree of bone marrow dysfunctioning, blood cell count will be collected in the database. Grading will be done according to several CTCAE v5.0 subcategories White blood cell decreased’, ‘Neutrophil count decreased’, ‘Anemia’ and ‘low platelets‘.

Extra procedures for this study are limited to questionnaires:

- Questionnaire EORTC QLQ-C305
- Questionnaire EORTC-QLQ-H&N3
- Questionnaire EQ-5D-5L
- Questionnaire Productivity Cost Analysis (PCQ) for low SMM patients only
- Semi structured interview 3 months after treatment

Skeletal muscle mass measurement

SMM will be measured on pre-treatment standard CT, MRI or PET CT, made during routine diagnostic work-up.

Cross-sectional muscle area (CSMA) is usually measured on a transversal single slice of the third lumbar vertebra (L3) leading to a lumbar skeletal muscle index (lumbar SMI) when corrected for stature.[40] This SMI correlates with total skeletal muscle volume measured on whole-body magnetic resonance imaging (MRI).[41]

In head- and neck cancer patients, head and neck CT-scan instead of an abdominal CT-scan is routinely performed.[2] However, by measuring the CSMA of the sternocleidomastoid muscles (SCM) and paravertebral muscles (PVM) on a transversal single slice of the third cervical vertebra (C3), SMM can be easily and reliably assessed.[35,36]

Muscle tissue was identified using Hounsfield Unit (HU) range settings from -29 to +150 HU, which is specific for muscle tissue. Muscle tissue was delineated at the level of the third cervical vertebra (C3). The SMA was defined as the pixel area within the delineated area with a radiodensity between -29 and +150 HU.[42] Delineation of muscle tissue was manually performed using the Slice-O-matic software v 5.0 (Tomovision, Canada). Muscle tissue delineation at the level of C3 was performed by selecting the first slide showing both transverse processes and the entire vertebral arc when scrolling from caudal to cranial direction. The contours of the paravertebral muscles and both sternocleidomastoid muscles were manually traced. The SMA at the level of C3 was calculated as the sum of the paravertebral muscle and both sternocleidomastoid muscles. If evident lymph node metastasis hindered accurate delineation of one sternocleidomastoid muscle, the SMA of the contralateral sternocleidomastoid muscle was used as an estimation of the SMA of the affected sternocleidomastoid muscle. After delineation, SMA was automatically retrieved from Slice-O-matic (v5.0, TomoVision, Montreal, Canada).[35] For MRI, muscle area was manually segmented, and fatty tissue was manually excluded. The overall intraclass correlation coefficient (ICC) for the muscle SMA obtained by CT and MRI has shown to be excellent (ICC 0.9, $p<0.01$), and can therefore be used interchangeably for measuring CSA at the level of C3.[43] The cervical SMI (CSMI) was calculated by dividing the SMA at the level of C3 by the squared height of the patient.

8.3.1 Study flowchart

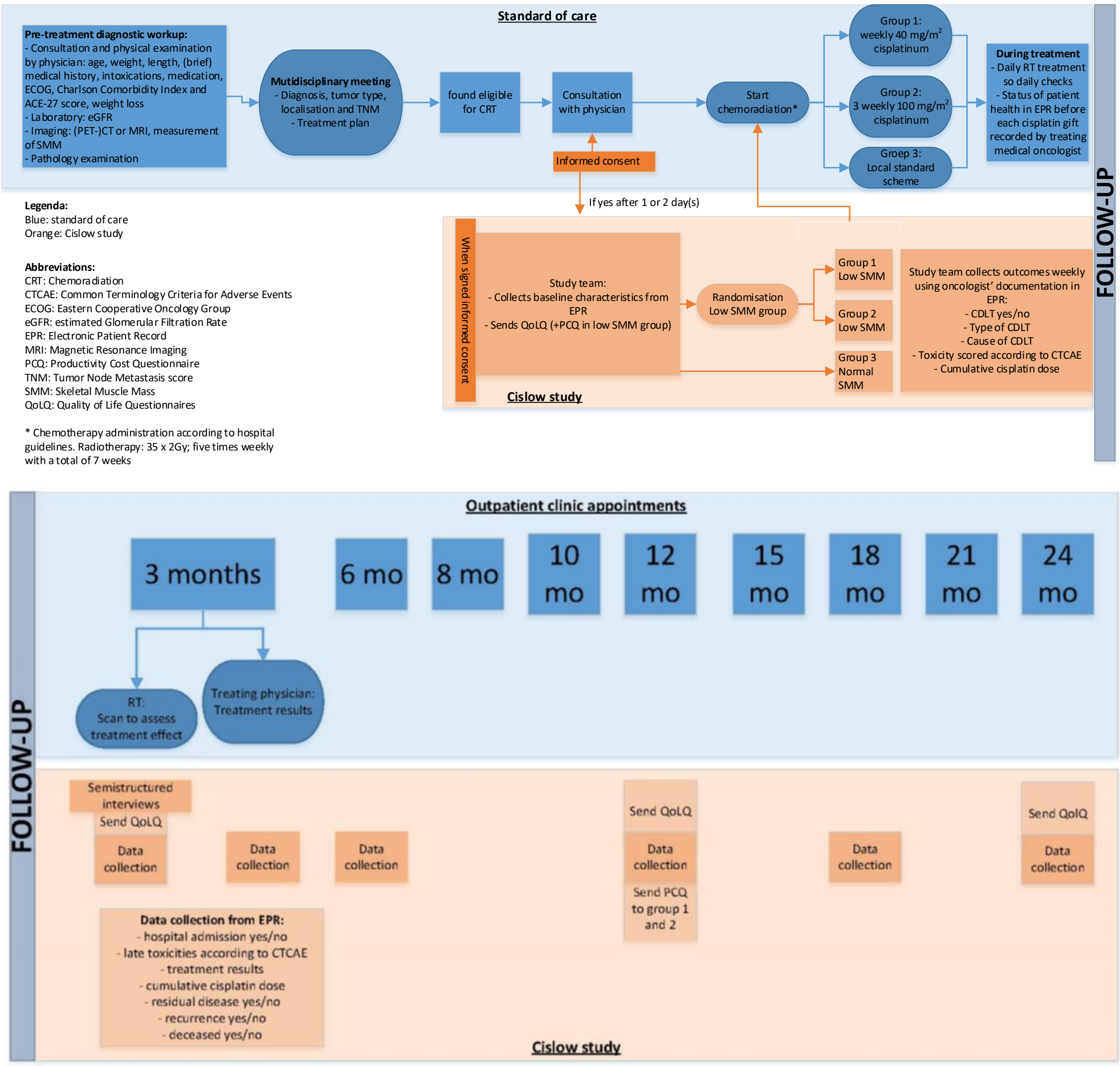

The end of treatment is defined as the day of the last radiation session. Follow-up time is calculated as time passed since the end of treatment. The first moment of follow-up is 3 months after end of treatment. For each follow-up moment, it is preferred to send the questionnaires and collect data as soon as possible, but a delay of maximum 6 weeks is also allowed and will not affect study progress.

8.4 Withdrawal of individual subjects

Subjects can leave the study at any time for any reason if they wish to do so without any consequences. The treating physician can decide to withdraw a subject from the study for urgent medical reasons.

8.4.1 Specific criteria for withdrawal (if applicable)

- The patient or its legal representative request that the patient will be withdrawn from the study.

- The sponsor decides to discontinue the study due to significant safety or efficacy
- concerns.
- The patient becomes pregnant.
- The treating physician withdraws the patient from the study. This decision can be made because of progressive disease, toxicity or patient's inability to be exposed to the study treatment. Since early termination is one of the outcomes of this study, we will not exclude the patient from data-analysis if not explicitly requested by the patient or treating physician.

8.5 Replacement of individual subjects after withdrawal

N.A.

8.6 Follow-up of subjects withdrawn from treatment

Follow-up consists of (clinical) examination according to the national guidelines for at least 24 months after end of treatment. The researcher will collect data as previously described. Follow-up data will be collected using EPR for withdrawn patients as well, when they were initially included, to prevent bias.

8.7 Premature termination of the study

This study will be terminated prematurely when new scientific evidence emerges making this study redundant.

9. SAFETY REPORTING

9.1 Temporary halt for reasons of subject safety

In accordance to section 10, subsection 4, of the WMO, the sponsor will suspend the study if there is sufficient ground that continuation of the study will jeopardise subject health or safety. The sponsor will notify the accredited METC without undue delay of a temporary halt including the reason for such an action. The study will be suspended pending a further positive decision by the accredited METC. The investigator will take care that all subjects are kept informed.

9.2 AEs, SAEs and SUSARs

9.2.1 Adverse events (AEs)

Adverse events are defined as any undesirable experience occurring to a subject during the study, whether or not considered related to the intervention. Adverse events classified according to the CTCAE criteria as grade 3 or higher, reported spontaneously by the subject or observed by the investigator or his/her staff and will be recorded. We do expect adverse events in this study, since this is common during cisplatin treatment, especially in a high-dose.[44,45]

9.2.2 Serious adverse events (SAEs)

A serious adverse event is any untoward medical occurrence or effect that occurs that:

- results in death;
- is life threatening (at the time of the event);
- requires extra hospitalisation or prolongation of existing inpatients' hospitalisation;
- results in persistent or significant disability or incapacity;
- is a congenital anomaly or birth defect; or
- any other important medical event that did not result in any of the outcomes listed above due to medical intervention but could have been, based upon appropriate judgement by the investigator.

The population of the present study consists of patients who are treated for HNSCC. Adverse events are relatively common in this population, and are expected to be related to their disease and treatment of the disease, not to participation in this study. Therefore the following events will not be reported as SAE and/or AE:

- an elective hospital admission;
- nausea (CTCAE v5.0 grade 1 through 3)
- mucositis (grade 1 through 3);
- acute kidney injury and/or electrolyte disbalances (grade 1 through 3);
- diarrhea (grade 1 through 3);

This study is expected to pose a negligible risk for patients; any serious adverse events occurring during the study period are expected to be related to the standard treatment and not to the study intervention. Because of this, SAEs that result in death or are life threatening will

be not reported expedited through the webportal Toetsingonline to the accredited METC that approved the protocol. The SAE's will be documented by the investigator and will be reported once a year.

9.2.3 Suspected unexpected serious adverse reactions (SUSARs)

Adverse reactions are all untoward and unintended responses to an investigational product related to any dose administered.

Unexpected adverse reactions are SUSARs if the following three conditions are met:

1. the event must be serious (see chapter 9.2.2);
2. there must be a certain degree of probability that the event is a harmful and an undesirable reaction to the medicinal product under investigation, regardless of the administered dose;
3. the adverse reaction must be unexpected, that is to say, the nature and severity of the adverse reaction are not in agreement with the product information as recorded in:
   - Summary of Product Characteristics (SPC) for an authorised medicinal product;

The sponsor will report expedited the following SUSARs through the web portal *ToetsingOnline* to the METC:

- SUSARs that have arisen in the clinical trial that was assessed by the METC;
- SUSARs that have arisen in other clinical trials of the same sponsor and with the same medicinal product, and that could have consequences for the safety of the subjects involved in the clinical trial that was assessed by the METC.

The remaining SUSARs are recorded in an overview list (line-listing) that will be submitted once every year to the METC. This line-listing provides an overview of these SUSARs from the study medicine, accompanied by a brief report highlighting the main points of concern. The expedited reporting of SUSARs through the web portal Eudravigilance or ToetsingOnline is sufficient as notification to the competent authority.

The sponsor will report expedited all SUSARs to the competent authorities in other Member States, according to the requirements of the Member States.

The expedited reporting will occur not later than 15 days after the sponsor has first knowledge of the adverse reactions. For fatal or life threatening cases the term will be maximal 7 days for a preliminary report with another 8 days for completion of the report.

9.3 Annual safety report

In addition to the expedited reporting of SUSARs, the sponsor will submit, once a year throughout the clinical trial, a safety report to the accredited METC, competent authority, and competent authorities of the concerned Member States.

This safety report consists of:

- a list of all suspected (unexpected or expected) serious adverse reactions of grade 3 or higher according to CTCAE v5 criteria, along with an aggregated summary table of all reported serious adverse reactions, ordered by organ system, per study. We will not report events that are mentioned in the SPC, since adverse events in cisplatin therapy are very common and and related to standard of care, not to this study;
- a report concerning the safety of the subjects, consisting of a complete safety analysis and an evaluation of the balance between the efficacy and the harmfulness of the medicine under investigation.

9.4 Follow-up of adverse events

All AEs of grade 3 or higher defined according to CTCAE criteria and not excluded by the list mentioned in 9.2.2, will be followed up by the researcher until they have abated, until a stable situation has been reached or until the study has ended. Depending on the event, follow up may require additional tests or medical procedures as indicated, and/or referral to the general physician or a medical specialist. Follow-up of adverse events will be according to standard hospital procedures. The investigator will collect the information using the EPR. SAEs need to be reported till end of study within the Netherlands, as defined in the protocol

9.5 [Data Safety Monitoring Board (DSMB) / Safety Committee]

NA, see 13.2

10. STATISTICAL ANALYSIS

Categorical data will be represented as a number and percentage of the total. Data analysis will be performed using statistical software R and SPSS Statistics. A test for normality (Kolmogorov-Smirnoff test) and histograms will be used to assess whether continuous variables are normally distributed. Continuous data will be represented as mean ± standard deviation (SD) if normally distributed, and median ± interquartile range (IQR) if skewed. Fisher's exact tests, Pearson Chi square tests, independent sample t-tests and Mann-Whitney U tests will be used to assess group differences. All analyses will be two-sided and $p<0.05$ is considered significant. Missing data will be handled using multiple imputation, if needed; we only expect few missing data to occur due to the nature and scale of the study.

10.1 Primary study parameter(s)

The primary outcome parameter is compliance (non CDLT) rate to the proposed cisplatin scheme in the intention-to-treat population.

10.2 Secondary study parameter(s)

Treatment toxicity is assessed with the Common Toxicity Criteria Adverse Events (CTCAE) v5.0 for a period of 24 months from the end of treatment. Toxicity scored as CDLT by the treating physician will be assessed, clarified if necessary, and reconfirmed separately. A severe toxic event is defined as a toxicity assessed as grade 3–5. Toxicities are classified as acute if they first appeared during or up to 3 months after treatment, and are classified as late if they persist, or first appear, more than 3 months after treatment. Multiple occurrences of events of a single toxicity type within an analysis time period are counted as a single event. Median numbers of toxicity events per patient (short-term [acute] toxicity and adverse long-term [late] effects, based on the TAME method of reporting toxicities) are compared between trial arms using t-tests. Proportions of patients affected by one or more toxicity event are compared between trial arms using Fishers exact test. Total administered cisplatin dose will also be recorded and means (or medians depending on the distribution) compared between trial arms.

Overall survival, disease-specific survival, loco-regional recurrences and time to recurrence are measured from the starting date of chemotherapy and compared between the three groups by the log-rank test with all-cause mortality in the intention-to treat population. Deaths are classified as being due to head and neck cancer or to other causes. Recurrences can be loco-regional or distant, or both. Patients who died from head and neck cancer causes as the first event are classed as recurrences. Patients on follow-up and patients lost to follow-up are censored at the last date at which they were known to be alive. Total time of follow-up per patient is two years. Hazard ratios and 95% CIs are generated with a univariate Cox proportional hazards model. The Schoenfeld residuals are tested by plotting the observed Kaplan- Meier values against the Cox predicted values. The effect of randomised treatment on outcome is also assessed after adjusting for known prognostic factors with a multivariate Cox proportional hazards model. At 3 months after treatment semi-structured interviews are conducted to obtain a better understanding of the patients' experience and preference for the treatment schemes, until saturation is reached. Semi-

structured interviews will be analyzed by two investigators using thematic descriptive analyses. Data coding will be done by open, axial and selective coding and will be supported by the software package NVivo.

10.3 Other study parameters

See 8.1.3.

10.4 Interim analysis (if applicable)

N.a.

11. ETHICAL CONSIDERATIONS

11.1 Regulation statement

This study will be conducted according to the principles of the Declaration of Helsinki (2013) and in accordance with the Medical Research Involving Human Subjects Act (WMO).

11.2 Recruitment and consent

Eligibility for this study will be assessed during the weekly multidisciplinary meeting where treatment plans of all new head and neck cancer patients are discussed. SMM will be measured on routinely performed (PET-)CT or MRI scans.

UMC Utrecht:
SMM will be measured by the treating physician (or the person who is delegated by the treating physician). Potentially eligible patients will be asked by the treating physician if they can be approached by a study-team member for information and patient information letter, during and/or after appointment with their physician. Screening for in- and exclusion criteria will be performed by the treating physician and study-team members. The patient will have at least one day, or as much time as needed, between receipt of the patient information letter and signing informed consent. The study-team member will sign both copies and return one copy to the patient and keeps one copy for administration.

VUmc, Noordwest Ziekenhuisgroep, Leiden University Medical Center and NKI-AVL:
Potentially eligible patients will be asked by the treating physician for information and patient information letter, during and/or after appointment with their physician. Screening for in- and exclusion criteria will be performed by the treating physician and study-team members. The patient will have at least one day, or as much time as needed, between receipt of the patient information letter and signing informed consent. The study-team member will sign both copies and return one copy to the patient and keeps one copy for administration. SMM will be measured after the patient has signed informed consent.

11.3 Objection by minors or incapacitated subjects (if applicable)

NA

11.4 Benefits and risks assessment, group relatedness

It is expected that patients with low skeletal muscle mass, might benefit from a low-dose weekly cisplatin regimen in terms of increased compliance and cumulative received cisplatin dose, less adverse events and improved quality of life. We think that the 40 mg/m^2 arm will show comparable side effects to the 100 mg/m^2 arm, however the hypothesis is that the frequency and extent of these side effects will decrease. Only questionnaires, which will take a small amount of time, will be the extra burden on both the intervention and control arm. No other diagnostics will be performed in comparison to standard treatment. We consider both schemes as clinical equipoise

according to expert opinion. In both schemes we aim to give a cumulative dose of more than 200 mg/m2 and both schemes are used in daily clinical practice.[16,21]
For the HNSCC patient group with low skeletal muscle mass, this study may serve as a basis for a personalized cisplatin dosing regimen in the future. This might result in less severe treatment related toxicity, less treatment de-escalation or termination, and possibly also a higher disease specific survival.

11.5 Compensation for injury

The sponsor/investigator has a liability insurance which is in accordance with article 7 of the WMO.

The sponsor (also) has an insurance which is in accordance with the legal requirements in the Netherlands (Article 7 WMO). This insurance provides cover for damage to research subjects through injury or death caused by the study. The insurance applies to the damage that becomes apparent during the study or within 4 years after the end of the study.

11.6 Incentives (if applicable)

There will be no special incentives or compensation for participants in this study. All study related measurements will be during or combined with regular scheduled hospital visits. In the unexpected event of the necessity of an additional visit to the hospital, directly attributed to study-participation, travel expenses are reimbursed.

12. ADMINISTRATIVE ASPECTS, MONITORING AND PUBLICATION

12.1 Handling and storage of data and documents

Data will be coded according the data management plan. The principal investigator, the study team, datamanagement and monitor will have access to the source data. The key to the code will be safeguarded by the study coordinators. Data will be kept 15 years. A web-based data management system (Castor EDC) will be used for data management. For exact details, please see the Datamanagement plan.

12.2 Monitoring and Quality Assurance

Monitoring is guaranteed by the UMC Utrecht by a centralised, independent monitor. A monitoring plan for research with negligible patient risk is submitted to the METC separately.

12.3 Amendments

Amendments are changes made to the research after a favourable opinion by the accredited METC has been given. All amendments will be notified to the METC that gave a favourable opinion.

12.4 Annual progress report

The sponsor/investigator will submit a summary of the progress of the trial to the accredited METC once a year. Information will be provided on the date of inclusion of the first subject, numbers of subjects included and numbers of subjects that have completed the trial, serious adverse events/ serious adverse reactions, other problems, and amendments.

12.5 Temporary halt and (prematurely) end of study report

The investigator/sponsor will notify the accredited METC and competent authority of the end of the study within a period of 8 weeks. The end of the study is defined as the last patient’s last follow-up moment (which is 24 months after the last day of radiation treatment).

The sponsor will notify the METC immediately of a temporary halt of the study, including the reason of such an action.

In case the study is ended prematurely, the sponsor will notify the accredited METC and competent authority within 15 days, including the reasons for the premature termination.

Within one year after the end of the study, the investigator/sponsor will submit a final study report with the results of the study, including any publications/abstracts of the study, to the accredited METC and competent authority.

12.5 Public disclosure and publication policy

We agree on the CCMO’s position on the disclosure/publication of the research results

obtained from studies involving human subjects. The research data will be disclosed unreservedly by the investigators.

13. STRUCTURES RISK ANALYSIS

13.1 Potential issues of concern

NA

13.2 Synthesis

Chapter 13.1 is skipped, since cisplatin is indicated in HNSCC and will be administered in dosage schemes that are both common in clinical practice and acknowledged by the National Comprehensive Cancer Network and European Society of Medical Oncology guidelines.[7,21] We would like to emphasize that both schemes can be considered as clinical equipoise according to expert opinion.[16,21] In both schemes we aim to give a cumulative dose of more than 200 mg/m^2 since it has been proven that patients with locally advanced HNSCC have a survival benefit when receiving cisplatin above 200mg/m^2.[16]

Assuming compliance in boths arms is 100%, we do not expect differences in outcomes for both treatment groups. However, patients who are not able to fulfill the proposed treatment schedule have disadvantage in terms of overall survival.[15,16,34] In a study by Wendrich et al. it was shown that people with low skeletal muscle mass had more often CDLT than people with a normal skeletal muscle mass (44.3% vs. 13.7%) and that patients with CDLT had a lower overall survival.[34] Since we do not know whether patients with low skeletal muscle mass will tolerate a weekly scheme better than a three-weekly scheme, we are not yet able to conclude which scheme is superior. It could be that patients tolerate the weekly scheme better, which gives them a higher chance of receiving more than 200 mg/m^2 cisplatinum and thus might improve survival rates. Regarding quality of life, it can also be that patients receiving the weekly scheme experience less cisplatinum-related side-effects which might have a positive effect at quality of life. Still, there is no evidence available so the previous stated hypotheses can also be true for the three-weekly dose.

Due to the toxic side effects of cisplatin and the vulnerability of HNSCC patients, we do expect SAE and AEs to occur in both study arms. However this is in line with common standard of care and will not be related to the study procedure, which makes this study a low intervention clinical trial that does not need a DSMB or safety committee.

14. REFERENCES

1. Bray F, Ferlay J, Soerjomataram I, Siegel RL, Torre LA, Jemal A. Global cancer statistics 2018: GLOBOCAN estimates of incidence and mortality worldwide for 36 cancers in 185 countries. *CA Cancer J Clin*. 2018;68(6):394-424. doi:10.3322/caac.21492
2. Grégoire V, Lefebvre JL, Licitra L, Felip E. Squamous cell carcinoma of the head and neck: EHNS-ESMO-ESTRO clinical practice guidelines for diagnosis, treatment and follow-up. *Ann Oncol*. 2010;21(SUPPL. 5):184-186. doi:10.1093/annonc/mdq185
3. Adelstein DJ, Li Y, Adams GL, et al. An intergroup phase III comparison of standard radiation therapy and two schedules of concurrent chemoradiotherapy in patients with unresectable squamous cell head and neck cancer. *J Clin Oncol*. 2003;21(1):92-98. doi:10.1200/JCO.2003.01.008
4. Forastiere AA, Goepfert H, Maor M, et al. Concurrent chemotherapy and radiotherapy for organ preservation in advanced laryngeal cancer. *N Engl J Med*. 2003;349(22):2091-2098. doi:10.1056/NEJMoa031317
5. Sher DJ, Adelstein DJ, Bajaj GK, et al. Radiation therapy for oropharyngeal squamous cell carcinoma: Executive summary of an ASTRO Evidence-Based Clinical Practice Guideline. *Pract Radiat Oncol*. 2017;7(4):246-253. doi:10.1016/j.prro.2017.02.002
6. Oosting SF, Haddad RI. Best practice in systemic therapy for head and neck squamous cell carcinoma. *Front Oncol*. 2019;9(AUG):1-9. doi:10.3389/fonc.2019.00815
7. Machiels JP, René Leemans C, Golusinski W, Grau C, Licitra L, Gregoire V. Squamous cell carcinoma of the oral cavity, larynx, oropharynx and hypopharynx: EHNS–ESMO–ESTRO Clinical Practice Guidelines for diagnosis, treatment and follow-up†. *Ann Oncol*. 2020;31(11):1462-1475. doi:10.1016/j.annonc.2020.07.011
8. Gebre-Medhin M, Brun E, Engström P, et al. ARTSCAN III: A randomized phase III study comparing chemoradiotherapy with cisplatin versus cetuximab in patients with locoregionally advanced head and neck squamous cell cancer. *J Clin Oncol*. 2021;39(1):38-47. doi:10.1200/JCO.20.02072
9. Pignon JP, Maître A le, Maillard E, Bourhis J. Meta-analysis of chemotherapy in head and neck cancer (MACH-NC): An update on 93 randomised trials and 17,346 patients. *Radiother Oncol*. 2009;92(1):4-14. doi:10.1016/j.radonc.2009.04.014
10. Calais G, Alfonsi M, Bardet E, et al. Randomized Trial of Radiation Therapy Versus. *J Natl Cancer Inst*. 1999;91(24):2081-2086.
11. Ghosh S, Rao PB, Kumar PR, Manam S. Concurrent chemoradiation with weekly cisplatin for the treatment of head and neck cancers: An institutional study on acute toxicity and response to treatment. *Asian Pacific J Cancer Prev*. 2015;16(16):7331-7335. doi:10.7314/APJCP.2015.16.16.7331
12. National Cancer Institute. US Department of Health and Human Services. Common Terminology Criteria for Adverse Events Version 5.0.
13. Rivelli TG, Mak MP, Martins RE, da Costa e Silva VT, de Castro G. Cisplatin based chemoradiation late toxicities in head and neck squamous cell carcinoma patients. *Discov Med*. 2015;20(108):57-66. http://europepmc.org/abstract/MED/26321088
14. Szturz P, Wouters K, Kiyota N, et al. Low-dose vs. high-dose cisplatin: Lessons learned from 59 chemoradiotherapy trials in head and neck cancer. *Front Oncol*. 2019;9(FEB). doi:10.3389/fonc.2019.00086
15. Strojan P, Vermorken JB, Beitler JJ, et al. Cumulative cisplatin dose in concurrent chemoradiotherapy for head and neck cancer: A systematic review. *Head & neck*. 2016;38 Suppl 1:E2151-8. doi:10.1002/hed.24026
16. Spreafico A, Huang S, Xu W, et al. Impact of cisplatin dose intensity on human papillomavirus-related and -unrelated locally advanced head and neck squamous cell carcinoma. *Eur J Cancer*. 2016;67:174.
17. Jacinto JK, Co J, Mejia MB, Regala EE. The evidence on effectiveness of weekly vs triweekly cisplatin concurrent with radiotherapy in locally advanced head and neck squamous cell carcinoma (HNSCC): a systematic review and meta-analysis. *Br J Radiol*. 2017;90(1079):20170442. doi:10.1259/bjr.20170442
18. Porceddu S V., Scotté F, Aapro M, et al. Treating Patients With Locally Advanced Squamous Cell Carcinoma of the Head and Neck Unsuitable to Receive Cisplatin-Based Therapy. *Front Oncol*. 2020;9(January):1-12. doi:10.3389/fonc.2019.01522
19. Medina JA, Rueda A, de Pasos AS, et al. A phase II study of concomitant boost radiation plus concurrent weekly cisplatin for locally advanced unresectable head and neck carcinomas.

Radiother Oncol. 2006;79(1):34-38. doi:10.1016/j.radonc.2006.03.010

20. Garden AS, Harris J, Vokes EE, et al. Preliminary results of Radiation Therapy Oncology Group 97-03: A randomized phase II trial of concurrent radiation and chemotherapy for advanced squamous cell carcinomas of the head and neck. *J Clin Oncol*. 2004;22(14):2856-2864. doi:10.1200/JCO.2004.12.012
21. National Comprehensive Cancer Network. NCCN Guidelines Version 1.2021 Head and neck cancers.
22. Noronha V, Joshi A, Patil VM, et al. Once-a-week versus once-every-3-weeks cisplatin chemoradiation for locally advanced head and neck cancer: a phase III randomized noninferiority trial. *J Clin Oncol*. 2018;36(11):1064-1072. doi:10.1200/JCO.2017.74.9457
23. Tsan D-L, Lin C-Y, Kang C-J, et al. The comparison between weekly and three-weekly cisplatin delivered concurrently with radiotherapy for patients with postoperative high-risk squamous cell carcinoma of the oral cavity. *Radiat Oncol*. 2012;7:215. doi:10.1186/1748-717X-7-215
24. Quon H, Leong T, Haselow R, Leipzig B, Cooper J, Forastiere A. Phase III study of radiation therapy with or without cis-platinum in patients with unresectable squamous or undifferentiated carcinoma of the head and neck: an intergroup trial of the Eastern Cooperative Oncology Group (E2382). *Int J Radiat Oncol Biol Phys*. 2011;81(3):719-725.
25. Ho KF, Swindell R, Brammer C V. Dose intensity comparison between weekly and 3-weekly Cisplatin delivered concurrently with radical radiotherapy for head and neck cancer: A retrospective comparison from New Cross Hospital, Wolverhampton, UK. *Acta Oncol (Madr)*. 2008;47(8):1513-1518. doi:10.1080/02841860701846160
26. Gupta T, Agarwal JP, Ghosh-Laskar S, Parikh PM, D'Cruz AK, Dinshaw KA. Radical radiotherapy with concurrent weekly cisplatin in loco-regionally advanced squamous cell carcinoma of the head and neck: a single-institution experience. *Head Neck Oncol*. 2009;1:17. doi:10.1186/1758-3284-1-17
27. Otty Z, Skinner MB, Dass J, et al. Efficacy and tolerability of weekly low-dose cisplatin concurrent with radiotherapy in head and neck cancer patients. *Asia Pac J Clin Oncol*. 2011;7(3):287-292. doi:10.1111/j.1743-7563.2011.01405.x
28. Rawat S, Srivastava H, Ahlawat P, et al. Weekly versus Three-Weekly Cisplatin-based Concurrent Chemoradiotherapy as definitive treatment in Head and Neck Cancer- Where do we stand? *Gulf J Oncolog*. 2016;1(21):6-11.
29. Traynor AM, Richards GM, Hartig GK, et al. Comprehensive IMRT plus weekly cisplatin for advanced head and neck cancer: the University of Wisconsin experience. *Head Neck*. 2010;32(5):599-606. doi:10.1002/hed.21224
30. Boulmay BC, Chera BS, Morris CG, et al. Definitive altered fractionation radiotherapy and concomitant weekly cisplatin for locally advanced head and neck cancer. *Am J Clin Oncol*. 2009;32(5):488-491. doi:10.1097/COC.0b013e318194f418
31. Laskar SG, Chaukar D, Deshpande M, et al. Phase III randomized trial of surgery followed by conventional radiotherapy (5 fr/Wk) (Arm A) vs concurrent chemoradiotherapy (Arm B) vs accelerated radiotherapy (6fr/Wk) (Arm C) in locally advanced, stage III and IV, resectable, squamous cell carcinoma o. *J Clin Oncol*. 2016;34(15_suppl):6004. doi:10.1200/JCO.2016.34.15_suppl.6004
32. Sharma A, Mohanti BK, Thakar A, Bahadur S, Bhasker S. Concomitant chemoradiation versus radical radiotherapy in advanced squamous cell carcinoma of oropharynx and nasopharynx using weekly cisplatin: A phase II randomized trial. *Ann Oncol*. 2010;21(11):2272-2277. doi:10.1093/annonc/mdq219
33. Federatie Medische Specialisten. Richtlijn Hoofd-halstumoren, chemoradiatie en bioradiatie van hoofd-hals. Richtlijnendatabase. Published 2014. https://richtlijnendatabase.nl/richtlijn/hoofd-halstumoren/chemoradiatie_en_bioradiatie_van_hoofd-hals.html
34. Wendrich AW, Swartz JE, Bril SI, et al. Low skeletal muscle mass is a predictive factor for chemotherapy dose-limiting toxicity in patients with locally advanced head and neck cancer. *Oral Oncol*. 2017;71:26-33. doi:10.1016/j.oraloncology.2017.05.012
35. Swartz JE, Pothen AJ, Wegner I, et al. Feasibility of using head and neck CT imaging to assess skeletal muscle mass in head and neck cancer patients. *Oral Oncol*. 2016;62:28-33. doi:10.1016/j.oraloncology.2016.09.006
36. Bril SI, Wendrich AW, Swartz JE, et al. Interobserver agreement of skeletal muscle mass measurement on head and neck CT imaging at the level of the third cervical vertebra. *Eur Arch Oto-Rhino-Laryngology*. 2019;276(4):1175-1182. doi:10.1007/s00405-019-05307-w
37. Kiyota N, Tahara M, Fujii H, et al. Phase II/III trial of post-operative chemoradiotherapy

comparing 3-weekly cisplatin with weekly cisplatin in high-risk patients with squamous cell carcinoma of head and neck (JCOG1008). *J Clin Oncol*. 2020;38(15_suppl):6502. doi:10.1200/JCO.2020.38.15_suppl.6502
38. Brierley J, Gospodarowicz M, Wittekind C. *TNM Classification of Malignant Tumours*. 8th ed. John Wiley; 2016.
39. Theunissen EAR, Dreschler WA, Latenstein MN, et al. A new grading system for ototoxicity in adults. *Ann Otol Rhinol Laryngol*. 2014;123(10):711-718. doi:10.1177/0003489414534010
40. Prado CMM, Lieffers JR, McCargar LJ, et al. Prevalence and clinical implications of sarcopenic obesity in patients with solid tumours of the respiratory and gastrointestinal tracts: a population-based study. *Lancet Oncol*. 2008;9(7):629-635. doi:10.1016/S1470-2045(08)70153-0
41. Shen W, Punyanitya M, Wang Z, et al. Total body skeletal muscle and adipose tissue volumes: estimation from a single abdominal cross-sectional image. *J Appl Physiol*. 2004;97(6):2333-2338. doi:10.1152/japplphysiol.00744.2004
42. Heymsfield SB, Wang Z, Baumgartner RN, Ross R. Human body composition: advances in models and methods. *Annu Rev Nutr*. 1997;17:527-558. doi:10.1146/annurev.nutr.17.1.527
43. Chargi N, Ansari E, Huiskamp LFJ, Bol G, de Bree R. Agreement between skeletal muscle mass measurements using computed tomography imaging and magnetic resonance imaging in head and neck cancer patients. *Oral Oncol*. 2019;99:104341. doi:https://doi.org/10.1016/j.oraloncology.2019.06.022
44. Blanchard P, Landais C, Petit C, et al. Meta-analysis of chemotherapy in head and neck cancer (MACH-NC): An update on 100 randomized trials and 19,248 patients, on behalf of MACH-NC group. *Ann Oncol*. 2016;27:vi328. doi:10.1093/annonc/mdw376.02
45. Sacco AG, Cohen EE. Current Treatment Options for Recurrent or Metastatic Head and Neck Squamous Cell Carcinoma. *J Clin Oncol Off J Am Soc Clin Oncol*. 2015;33(29):3305-3313. doi:10.1200/JCO.2015.62.0963
